# Requirement for Cyclin D1 Underlies Cell-Autonomous HIF2 Dependence in Kidney Cancer

**DOI:** 10.1158/2159-8290.CD-24-1378

**Published:** 2025-04-04

**Authors:** Nitin H. Shirole, Devishi Kesar, Yenarae Lee, Amy Goodale, Sudeepa Syamala, Shweta Kukreja, Rong Li, Xintao Qiu, Wenyu Yu, Seth Goldman, Paloma Cejas, Henry W. Long, Karen Adelman, John G. Doench, William R. Sellers, William G. Kaelin

**Affiliations:** 1 Department of Medical Oncology, Dana-Farber Cancer Institute, Harvard Medical School, Boston, Massachusetts.; 2 Broad Institute of Massachusetts Institute of Technology and Harvard, Cambridge, Massachusetts.; 3 Center for Functional Cancer Epigenetics, Dana-Farber Cancer Institute, Harvard Medical School, Boston, Massachusetts.; 4 Nascent Transcriptomics Core, Harvard Medical School, Boston, Massachusetts.; 5 Department of Biological Chemistry and Molecular Pharmacology, Harvard Medical School, Boston, Massachusetts.; 6 Department of Medicine, Brigham and Women’s Hospital, Boston, Massachusetts.; 7 Howard Hughes Medical Institute, Chevy Chase, Maryland.

## Abstract

Inactivation of the *VHL* gene stabilizes HIF2α, which drives clear-cell renal cell carcinoma (ccRCC). The HIF2α inhibitor belzutifan is approved for ccRCC treatment, but *de novo* and acquired resistance are common. HIF2α, bound to ARNT, transcriptionally activates many genes. We performed CRISPR-mediated gene activation screens in HIF2α-dependent ccRCC lines treated with a belzutifan analog to identify HIF2α-responsive genes that confer cell-autonomous belzutifan resistance when not downregulated. Sustaining the expression of the HIF2α target gene *CCND1*, encoding cyclin D1, promoted HIF2α independence/belzutifan resistance. This activity requires CDK4/6 activation by cyclin D1 but is not solely due to phosphorylation of the canonical cyclin D1 target, pRB. Indeed, ccRCC lines lacking all three pRB family members remained at least partially HIF2α-dependent. In this context, however, a kinase-defective cyclin D1 variant partially overrode belzutifan’s antiproliferative effects, suggesting that ccRCC promotion by cyclin D1 requires the phosphorylation of pRB paralogs and one or more kinase-independent cyclin D1 activities.

**Significance:**

We discovered that cyclin D1 is the key target of HIF2 driving the cell-autonomous proliferation of *VHL*-mutant kidney cancers and that cyclin D1 has targets beyond pRB in this setting. These findings have implications for treating kidney cancer with HIF2 inhibitors, alone or in combination with CDK4/6 inhibitors.

## Teaser

The cell-intrinsic antiproliferative effects of HIF2 inhibitors in kidney cancer are caused by loss of cyclin D1 activity, and upregulation of cyclin D2 or D3 are potential resistance mechanisms.

## Introduction

Germline mutations that inactivate the *VHL* tumor-suppressor gene cause von Hippel–Lindau disease, which predisposes individuals to the most common form of kidney cancer, clear-cell renal cell carcinoma (ccRCC), as well as various other tumors such as hemangioblastomas and paragangliomas (1, 2). Tumor development in this setting is caused by the loss of the remaining wild-type (WT) *VHL* allele as well as by ensuing mutations in other genes (3, 4). Consistent with this knowledge, biallelic inactivation of the *VHL* tumor-suppressor gene is also the initiating event in most sporadic ccRCCs (5, 6).

The *VHL* gene product, pVHL, is the substrate recognition unit of an ubiquitin ligase that targets the α subunit of the heterodimeric HIF transcription factor for degradation when oxygen is plentiful (7). The inappropriate accumulation of HIF2, consisting of HIF2α and ARNT, drives the formation of ccRCC (5, 6).

HIF2α can be inhibited with small-molecule allosteric inhibitors that prevent it from binding to ARNT (8–10). Such inhibitors are highly active against ccRCC both preclinically and clinically (5, 11–18). One of these molecules, belzutifan, has now been approved by the FDA for the treatment of von Hippel–Lindau disease (19) and is approved for sporadic ccRCC based on a recently completed phase 3 trial (18).

HIF2 transcriptionally regulates hundreds of genes, many of which are known or suspected of being protumorigenic, including the gene encoding the secreted growth factor VEGF (16). Drugs that block VEGF or its receptor are now mainstays of ccRCC treatment (5). Genetic disruption of *EPAS1*, which encodes HIF2α, with RNAi or CRISPR-based gene editing impairs the fitness of pVHL-defective ccRCC lines in cellular competition assays, suggesting that HIF2α has both cell-autonomous and nonautonomous effects on ccRCC (20–24).

In the simplest view, the cell-autonomous effects of inhibiting HIF2 would reflect the suppression of a handful of key HIF2 target genes. If true, preventing their suppression would at least partially mitigate the effects of HIF2 inhibitors on ccRCC fitness. We therefore used CRISPR-mediated gene activation (CRISPRa) technology to identify HIF2 target genes that, when activated, conferred resistance to PT2399, reasoning that such genes would play critical roles in determining the clinical outcomes of HIF2 blockade and provide new insights into the mechanism of action of HIF2 inhibitors.

## Results

OSRC2 and TUHR4TKB renal carcinoma cell lines are examples of pVHL-defective ccRCC lines that display a proliferative disadvantage after CRISPR-mediated inactivation of *EPAS1* in competition assays carried out over weeks *in vitro* (21, 24). In pilot experiments, we asked whether these cells were amenable to CRISPRa using a system described by Sanson and Doench (25). Toward this end, the cells were engineered to stably express a nuclease-dead Cas9 (dCas9) fused to the VP64 transactivation domain and then stably infected with a lentiviral vector encoding (i) a modified single-guide RNA (sgRNA) containing two PP7 aptamers and two MS2 aptamers and (ii) phage coat protein fused to p65 and heat shock factor transactivation domains.

**Figure 1.**
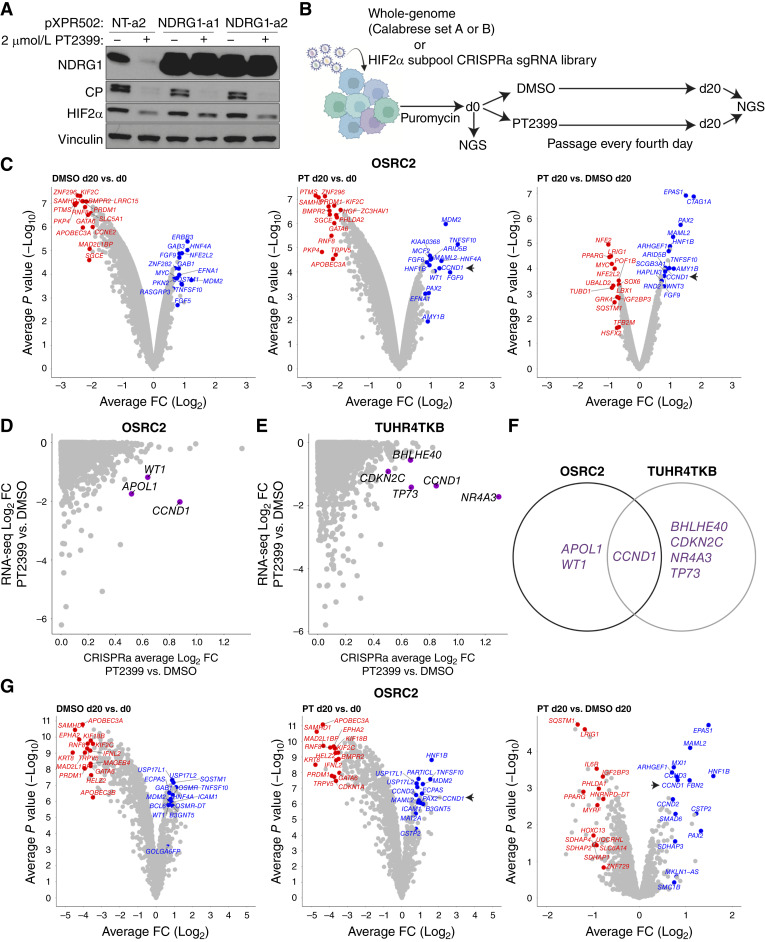
CRISPRa screens for modulators of ccRCC sensitivity to HIF2α inhibition. **A,** Immunoblot analysis of OSRC2 cells expressing dCas9-VP64 that were infected with indicated CRISPRa sgRNAs and treated with 2 μmol/L of the HIF2α inhibitor PT2399 or DMSO for 4 days. **B,** Schematic of CRISPRa screens performed in HIF2α-dependent ccRCC cells that were either infected with whole-genome or HIF2α subpool (see text) CRISPRa sgRNA libraries in the presence or absence of the 2 μmol/L PT2399. **C,** Volcano plots showing genes whose sgRNAs were enriched or depleted in DMSO d20 vs. d0 (left), PT2399 (PT) d20 vs. d0 (middle), and PT d20 vs. DMSO d20 (right) in OSRC2 cells expressing dCas9-VP64 that were infected with the whole-genome CRISPRa library. The top 15 genes based on the average FC (log 2) whose sgRNAs were enriched (blue) or depleted (red) are labeled. Arrowheads indicate the location of *CCND1* on the volcano plots. *n* = 2 biological replicates. **D,** Scatterplots of genes whose sgRNAs were enriched in the PT2399 vs. DMSO arm of CRISPRa screens (*x*-axis) in OSRC2 cells compared with genes whose expression was downregulated by treatment with PT2399 for 24 hours (*y*-axis). Genes with CRISPRa average log_2_ FC > 0.5 and *P* value < 0.05 and RNA-seq average log_2_ FC < 0.5 and *P* value < 0.05 were labeled and colored purple. **E,** Scatterplots of genes whose sgRNAs were enriched in PT2399 vs. DMSO arm of CRISPRa screens (*x*-axis) in TUHR4TKB cells compared with genes whose expression was downregulated by treatment with PT2399 for 24 hours (*y*-axis). Genes with CRISPRa average log_2_ FC > 0.5 and *P* value < 0.05 and RNA-seq average log_2_ FC < 0.5 and *P* value < 0.05 were labeled and colored purple. **F,** Venn diagram of showing overlap between the genes from OSRC2 (**D**) and TUHR4TKB (**E**) cells. **G,** Volcano plots showing genes whose sgRNAs were enriched or depleted in DMSO d20 vs. d0 (left), PT2399 (PT) d20 vs. d0 (middle), and PT d20 vs. DMSO d20 (right) in OSRC2 cells expressing dCas9-VP64 that were infected with the HIF2α subpool CRISPRa library. The top 15 genes based on the average FC (log 2) whose sgRNAs were enriched (blue) or depleted (red) are labeled. Arrowheads indicate the location of *CCND1* on the volcano plots. *n* = 2 biological replicates.

The HIF2-responsive gene products NDRG1 and ceruloplasmin were, as expected, downregulated by the HIF2α inhibitor PT2399 in cells that received a control sgRNA (NT-a2; Fig. 1A; Supplementary Fig. S1A). In contrast, NDRG1 protein levels were sustained in cells that received either of the two different *NDRG1* CRISPRa sgRNAs (Fig. 1A; Supplementary Fig. S1A). This was specific because the NDRG1 sgRNAs did not rescue the expression of ceruloplasmin nor of HIF2α itself, which is often modestly downregulated by PT2399-like chemicals (Fig. 1A; Supplementary Fig. S1A). The latter likely reflects the stability of unbound HIF2α compared with HIF2α bound to ARNT.

Next the OSRC2 and TUHR4TKB cells stably expressing dCas9-VP64 were infected with a genome-wide CRISPRa sgRNA library (Calabrese sets A and B) that also conferred puromycin resistance (Fig. 1B; Supplementary Fig. S1B). After 4 days of selection with puromycin, an aliquot of cells was removed for isolation of genomic DNA [day 0 (d0)]. The remaining cells were then split and treated with PT2399 or DMSO. The cells were passaged every 4 days and harvested for genomic DNA on day 20 (d20).

We noted enrichment over time of the sgRNAs activating *c-MYC*, *SQSTM1*, and *NFE2L2* (d20 compared with d0) in the OSRC2 cells treated with DMSO (Fig. 1C, left). c-*MYC* and *SQSTM1* are suspected of being driver oncogenes within the 8q and 5q recurrent amplicons in ccRCC, respectively (26, 27). *SQSTM1* encodes p62, which activates the *NFE2L2* gene product NRF2 (28). These sgRNAs, as well as sgRNAs for *NFE2*, were far less enriched in the PT2399-treated cells (d20 compared with d0) relative to the DMSO-treated cells, such that they seemed to be depleted when comparing the PT2399 d20 samples to the DMSO d20 samples (Fig. 1C, middle and right). These findings are consistent with c-*MYC* (29) and NRF2 cooperating with HIF2α to promote OSRC2 ccRCC fitness. *c-MYC*, but not *SQSTM1* and *NFE2L2*, scored similarly in the TUHR4TKB cells (Fig. S1C, right).

We next focused on genes whose sgRNAs were enriched after 20 days of PT2399 treatment compared with 20 days of DMSO treatment, reasoning that normalizing to the latter would at least partially eliminate genes that promoted growth irrespective of treatment rather than specifically engendering resistance to PT2399 (Fig. 1C; Supplementary Fig. S1C, right). To enrich for HIF2-responsive genes, we treated OSRC2 and TUHR4TKB cells with PT2399 for 24 hours (Fig. 1D and E) or 48 hours (Supplementary Fig. S1D and S1E), performed RNA sequencing (RNA-seq; Fig. S2A-B), and looked for genes that were enriched in the CRISPRa screen [log fold change (LFC) > 0.5 and *P* < 0.05] and transcriptionally downregulated by PT2399 (LFC < −0.5 and *P* < 0.05) at either or both time points. *CCND1*, *APOL1*, *MAP3K8*, *NR4A1*, and *WT1* met these two criteria in OSCR2 cells and *CCND1*, *BHLHE40*, *CCNE1*, *CDKN2C*, *MAP3K21*, *MYBL1*, *NR4A3*, *SSC5D*, and *TP73* in TUHR4TKB cells. Therefore, *CCND1* was the only gene that scored in both cells (Fig. 1D–F; Supplementary Fig. S1D–S1F).

To further address the reproducibility and robustness of our findings, we made a custom CRISPRa sgRNA library (“HIF2α subpool sgRNA library”) that targets 936 genes with ∼12 sgRNAs per gene. The 936 genes include (i) 294 genes whose CRISPRa guides were enriched in the PT2399-treated arm of the OSRC2 whole-genome CRISPRa screen (LFC > 0.5 and *P* < 0.05), (ii) 293 genes whose CRISPRa guides were depleted in the PT2399-treated arm of the OSRC2 whole-genome CRISPRa screen (LFC < −0.5 and *P* < 0.05), and (iii) 282 genes whose expression decreased after PT2399 in RNA-seq and precision run-on sequencing (PRO-seq) datasets (LFC < −0.5 and *P* < 0.05). The library also targets other known HIF2 regulators [e.g., *HIF1β* (encoding ARNT), *EP300*, *TRAPP*, and *TRIM22*] and ccRCC oncogenes (e.g., *PAX8* and *SQSTM1*; See Supplementary Table S1). The library, which also contains 300 nontargeting guides as controls, was then used to screen OSRC2 (Fig. 1B), TUHR4TKB (Supplementary Fig. S1B), and 786-O (Supplementary Fig. S3A and S3B) cells that were engineered to express dCas9-VP64 and treated with PT2399 or DMSO as described above. *CCND1* was the top-scoring gene based on sgRNA enrichment in the PT2399-treated 786-O (Supplementary Fig. S3C) cells compared with the DMSO-treated 786-O cells and again rescored positively in the OSRC2 cells and TUHR4TKB cells (Fig. 1G; Supplementary Fig. S1G). Interestingly, the *CCND1* paralog *CCND2*, which is not normally expressed in TUHR4TKB cells, was the top-scoring gene in these cells (Supplementary Fig. S1G).

**Figure 2.**
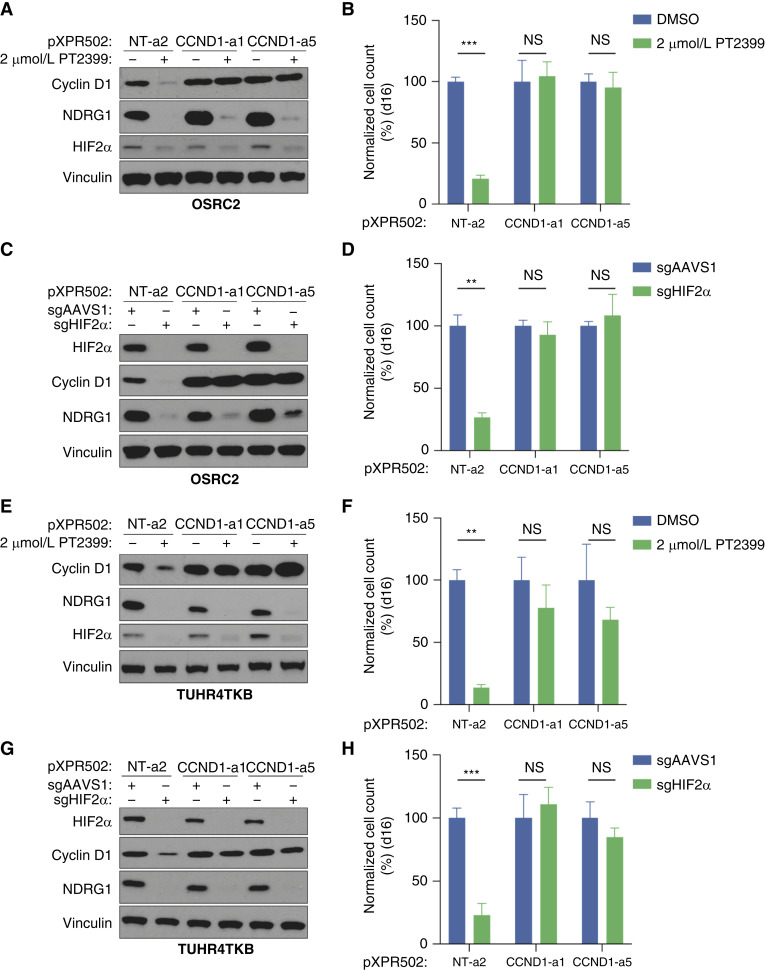
Failure to downregulate *CCND1* confers resistance to HIF2α inhibition. **A,** Immunoblot analysis of OSRC2 cells expressing dCas9-VP64 that were infected with indicated CRISPRa sgRNAs and treated with 2 μmol/L PT2399 or DMSO for 4 days. **B,** Cellular proliferation assays of cells as in (**A**) that were treated with 2 μmol/L PT2399 or DMSO for 16 days. Data are the means ± SD of *n* = 3 biological replicates and are normalized to the DMSO-treated cells for each respective sgRNA. ***, *P* < 0.001, and NS (not significant), Unpaired *t* test. **C,** Immunoblot analysis of OSRC2 cells expressing dCas9-VP64 that were infected with indicated CRISPRa sgRNAs and subsequently nucleofected with RNPs containing Cas9 and either sgAAVS1 or sgHIF2α. **D,** Cellular proliferation assays of cells as in **C**. Data are the means ± SD of *n* = 3 biological replicates and are normalized to the sgAAVS1 cells for each of the respective CRISPRa sgRNAs. **, *P* < 0.01, and NS, unpaired *t* test. **E,** Immunoblot analysis of TUHR4TKB cells expressing dCas9-VP64 that were infected with indicated CRISPRa sgRNAs and treated with 2 μmol/L PT2399 or DMSO for 4 days. **F,** Cellular proliferation assays of cells as in (**E**) that were treated with 2 μmol/L PT2399 or DMSO for 16 days. Data are the means ± SD of *n* = 3 biological replicates and are normalized to the DMSO-treated cells for each respective sgRNA. **, *P* < 0.01, and NS, unpaired *t* test. **G,** Immunoblot analysis of TUHR4TKB cells expressing dCas9-VP64 that were infected with indicated CRISPRa sgRNAs and subsequently nucleofected with RNPs containing Cas9 and either sgAAVS1 or sgHIF2α. **H,** Cellular proliferation assays of cells as in **G**. Data are the means ± SD of *n* = 3 biological replicates and are normalized to the sgAAVS1 cells for each of the respective CRISPRa sgRNAs. ***, *P* < 0.001, and NS, unpaired *t* test.

**Figure 3.**
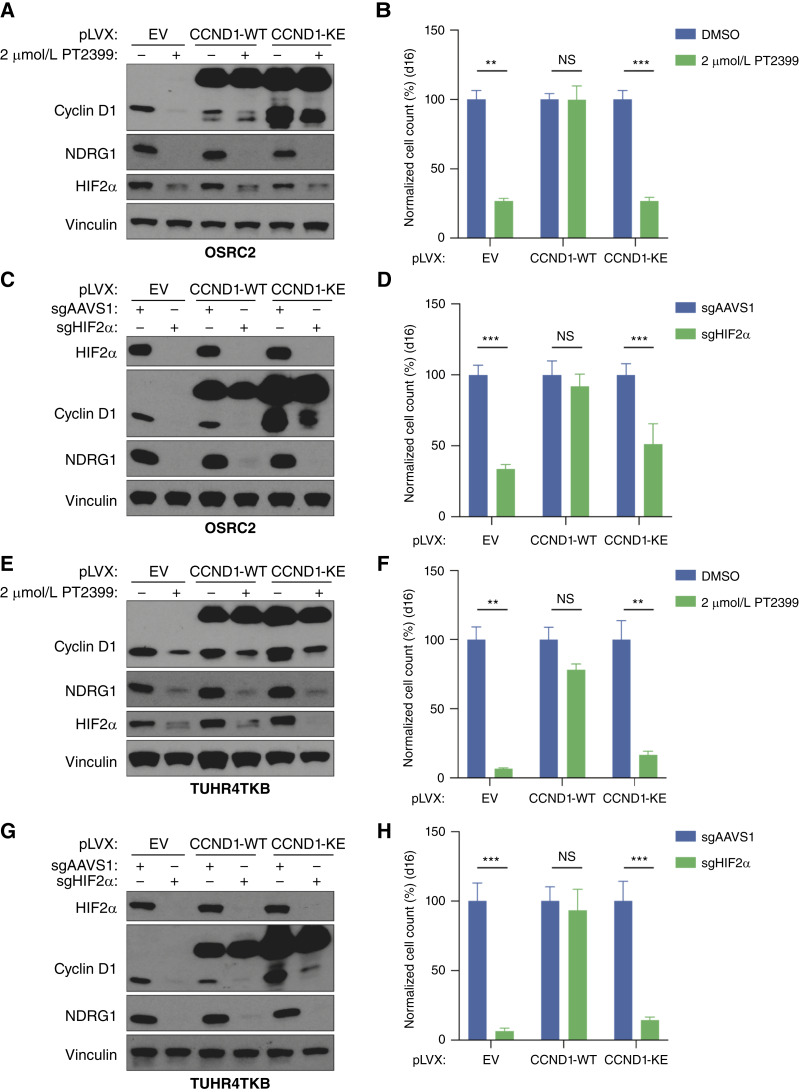
Cyclin D1 kinase activity is required for cyclin D1 to confer HIF2α independence **A,** Immunoblot analysis of OSRC2 cells stably expressing cyclin D1 (WT or K112E) or the EV and treated with 2 μmol/L PT2399 or DMSO for 4 days. **B,** Cellular proliferation assays of cells as in (**A**) that were treated with 2 μmol/L PT2399 or DMSO for 16 days. Data are the means ± SD of *n* = 3 biological replicates and were normalized to the DMSO-treated cells for the respective cell lines (EV, WT, or KE). **, *P* < 0.01; ***, *P* < 0.001, and NS (not significant), unpaired *t* test. **C,** Immunoblot analysis of OSRC2 cells stably expressing cyclin D1 (WT or K112E) or the EV and subsequently nucleofected with RNPs containing Cas9 and either sgAAVS1 or sgHIF2α. **D,** Cellular proliferation assays of cells as in **C**. Data are the means ± SD of *n* = 3 biological replicates and were normalized to the sgAAVS1 cells for the respective cell lines (EV, WT, or KE). ***, *P* < 0.001, and NS, unpaired *t* test. **E,** Immunoblot analysis of TUHR4TKB cells stably expressing cyclin D1 (WT or K112E) or the EV and treated with 2 μmol/L PT2399 or DMSO for 4 days. **F,** Cellular proliferation assays of cells as in (**E**) that were treated with 2 μmol/L PT2399 or DMSO for 16 days. Data are the means ± SD of *n* = 3 biological replicates and were normalized to the DMSO-treated cells for the respective cell lines (EV, WT, or KE). **, *P* < 0.01, and NS, unpaired *t* test. **G,** Immunoblot analysis of TUHR4TKB cells stably expressing cyclin D1 (WT or K112E) or the EV and subsequently nucleofected with RNPs containing Cas9 and either sgAAVS1 or sgHIF2α. **H,** Cellular proliferation assays of cells as in **G**. Data are the means ± SD of *n* = 3 biological replicates and were normalized to the sgAAVS1 cells for the respective cell lines (EV, WT, or KE). ***, *P* < 0.001, and NS, unpaired *t* test.

*CCND1* is upregulated by HIF2 in pVHL-defective ccRCCs, and a *CCND1* enhancer contains a HIF2-binding site (30–32). We confirmed by chromatin immunoprecipitation sequencing (ChIP-seq; Supplementary Fig. S4A) and PRO-seq (Supplementary Fig. S4B) that *CCND1* is a direct transcriptional target of HIF2α in OSRC2 cells. We also confirmed that preventing the downregulation of *CCND1* with two different *CCND1* CRISPRa sgRNAs (Fig. 2A–H; Supplementary Fig. S5A–S5D), or by exogenous *CCND1* expression driven by a constitutive promoter (Fig. 3A–H; Supplementary Fig. S6A–S6D), rendered ccRCC lines largely resistant to PT2399 and to CRISPR-mediated elimination of HIF2α.

Cyclin D1 forms complexes with CDK4 or CDK6 that phosphorylate specific proteins linked to cell-cycle progression such as the retinoblastoma tumor suppressor protein (pRB; refs. 33, 34). Exogenous expression of WT cyclin D1, but not a kinase-defective cyclin D1 mutant (K112E; ref. 35), conferred resistance to PT2399 and to HIF2α loss (Fig. 3A–H; Supplementary Fig. S6A–S6D). Decreased cell number after PT2399 treatment was associated with impaired G1/S traversal, as determined by cell-cycle FACS (Supplementary Fig. S7A and S7B) and EdU incorporation (Supplementary Fig. S7C and S7F), and not apoptosis, as determined by annexin V staining (Supplementary Fig. S8A and S8B), consistent with a role for cyclin D1.

**Figure 4.**
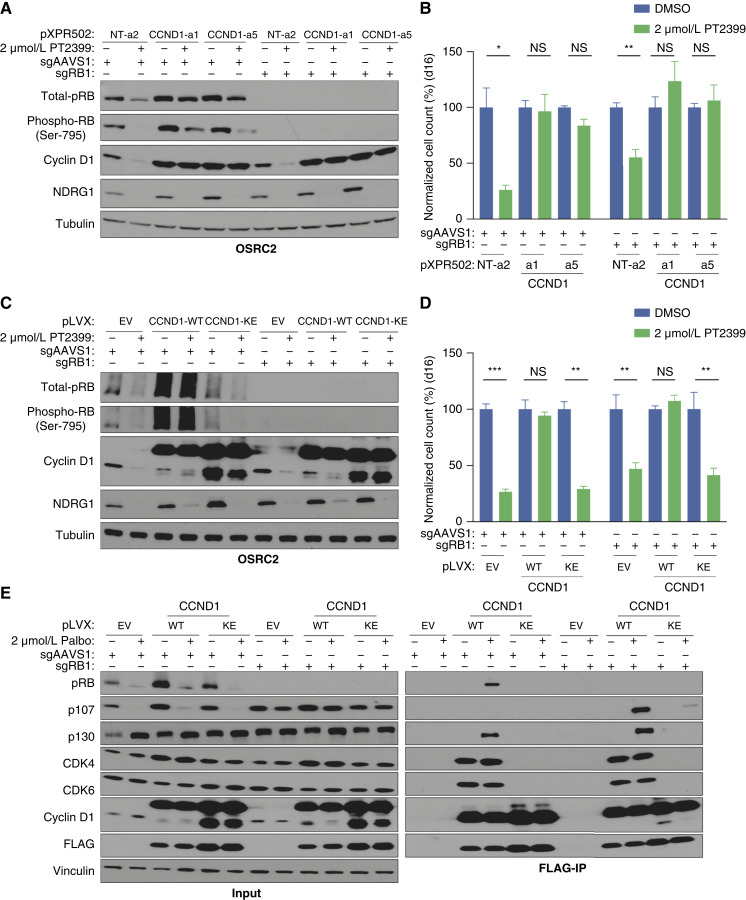
Inactivation of pRB is not sufficient to confer HIF2α independence: potential roles of pRB paralogs as cyclin D1 targets. **A,** Immunoblot analysis of OSRC2 cells expressing dCas9-VP64 that were infected with indicated CRISPRa sgRNAs, nucleofected with RNPs containing Cas9 and either sgAAVS1 or sgRB1, and then treated with 2 μmol/L PT2399 or DMSO for 4 days. **B,** Cellular proliferation assays of cells as in (**A**) that were treated 2 μmol/L PT2399 or DMSO for 16 days. Data are the means ± SD of *n* = 3 biological replicates and were normalized to the DMSO-treated cells for the respective CRISPRa sgRNAs. *, *P* < 0.05; **, *P* < 0.01; and NS, unpaired *t* test. **C,** Immunoblot analysis of OSRC2 cells stably expressing cyclin D1 (WT or K112E) or the EV that were nucleofected with RNPs as in (**A**) and then treated with 2 μmol/L PT2399 or DMSO for 4 days. **D,** Cellular proliferation assays of cells as in (**C**) that were treated with 2 μmol/L PT2399 or DMSO for 16 days. Data are the means ± SD of *n* = 3 biological replicates and were normalized to the DMSO-treated cells for the respective cell lines (EV, WT, or KE). **, *P* < 0.01; ***, *P* < 0.001; and NS, unpaired *t* test. **E,** Immunoblot analysis of input (left) and anti-FLAG immunoprecipitates (right) from OSRC2 stably expressing cyclin D1 (WT or K112E) or the EV that were nucleofected with RNPs containing Cas9 and either sgAAVS1 or sgRB1. The cells were then treated with 2 μmol/L palbociclib or DMSO, where indicated, for 16 hours to promote substrate-trapping. NS, not significant; phospho = phosphorylated.

Loss of pRB abrogates the antiproliferative effects of CDK4/6 inhibitors in most preclinical models (36, 37). Surprisingly, CRISPR-mediated inactivation of *RB1*, which encodes pRB, did not eliminate the sensitivity of OSRC2 and TUHR4TKB cells to PT2399 (Fig. 4A–D; Supplementary Fig. S9A–S9D). To begin to understand this, we stably infected *RB1+/+* and *RB1−/−* OSRC2 cells with lentiviruses expressing FLAG epitope–tagged versions of cyclin D1 WT, cyclin D1 K112E, or the empty vector (EV), treated them with the CDK4/6 inhibitor palbociclib or DMSO, and performed immunoprecipitations followed by Western blot analysis. Anti-FLAG immunoprecipitation of WT cyclin D1, but not cyclin D1 K112E, from the *RB1+/+* cells captured pRB and the pRB paralog p130 in cells treated with palbociclib, consistent with palbociclib converting cyclin D1 complexes into substrate traps (Fig. 4E). Notably, anti-FLAG immunoprecipitation of cyclin D1 from *RB1*−*/*− cells recovered p130 and the pRB paralog p107, suggesting that p107 can substitute for pRB to suppress cell proliferation in *RB1*−*/*− ccRCC cells deprived of CDK4/6 activity (Fig. 4E).

**Figure 5.**
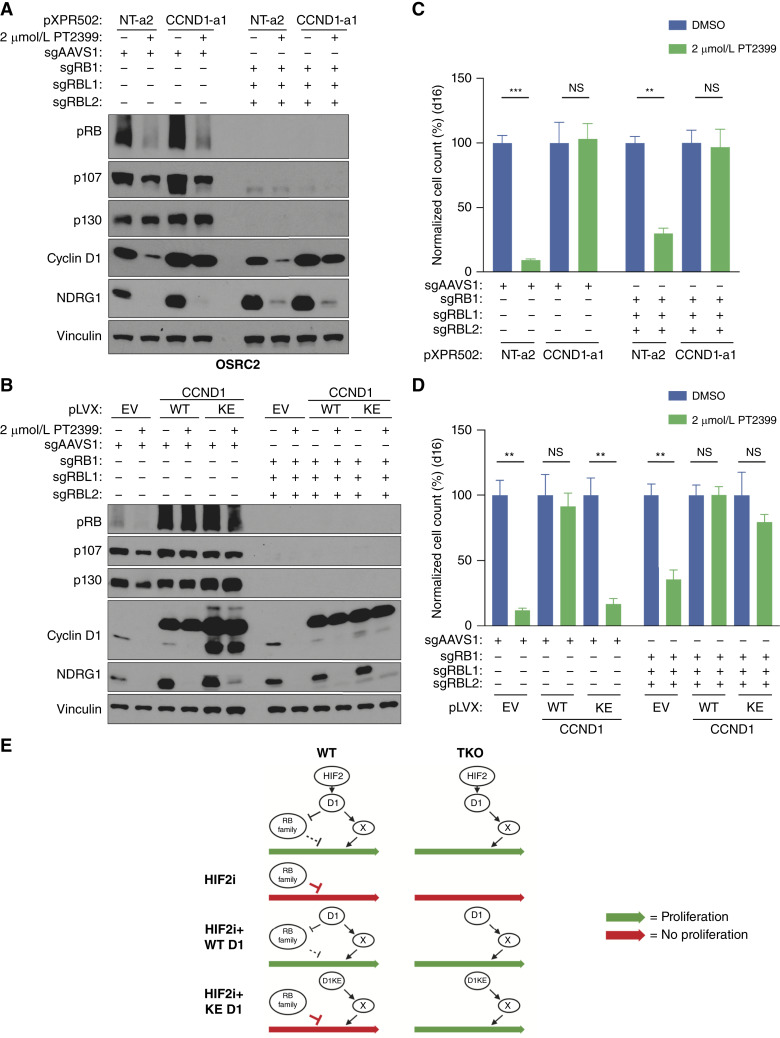
Cyclin D1 kinase activity is dispensable for cyclin D1 to confer HIF2α independence in the cells lacking all three pRB paralogs. **A,** Immunoblot analysis of OSRC2 cells expressing dCas9-VP64 that were infected with indicated CRISPRa sgRNAs, nucleofected with RNPs containing Cas9 and the indicated sgRNAs, and then treated with 2 μmol/L PT2399 or DMSO for 4 days. **B,** Immunoblot analysis of OSRC2 cells stably expressing cyclin D1 (WT or K112E) or the EV that were nucleofected with RNPs containing Cas9 and the indicated sgRNAs and then treated with 2 μmol/L PT2399 or DMSO for 4 days. **C,** Cellular proliferation assays of cells as in (**A**) that were treated with 2 μmol/L PT2399 or DMSO for 16 days. Data are the means ± SD of *n* = 3 biological replicates and were normalized to the DMSO-treated cells for the respective CRISPRa sgRNAs. **, *P* < 0.01; ***, *P* < 0.001; and NS, unpaired *t* test. **D,** Cellular proliferation assays of cells as in (**B**) that were treated with 2 μmol/L PT2399 or DMSO for 16 days. Data are the means ± SD of *n* = 3 biological replicates and were normalized to the DMSO-treated cells for the respective cell lines (EV, WT, or KE). **, *P* < 0.01, and NS, unpaired *t* test. **E,** Model for unmasking of kinase-independent cyclin D1 activities in cells lacking all three RB family members. NS, not significant.

To test this, we nucleofected OSRC2 and TUHR4TKB cells with recombinant Cas9 and sgRNAs designed to inactivate *RB1*, *RBL1* (encoding p107), and *RBL2* (encoding p130) and confirmed gene inactivation by immunoblot analysis (Fig. 5A and B; Supplementary Figs. S10A, S10B, S11A, and S11B) and amplicon sequencing (Supplementary Fig. S12A–S12D) of the resulting polyclonal cell population. None of these three genes is essential in ccRCC, and the *RB1* paralog triple knockout (TKO) cells, if anything, grew faster than the parental cells. Remarkably, TKO OSRC2 and TUHR4TKB cells remained at least partially sensitive to PT2399 (Fig. 5C and D; Supplementary Figs. S10C, S10D, S11C, and S11D). This residual sensitivity suggests the existence of a cyclin D1 substrate outside the pRB family, or perhaps a noncanonical cyclin D1 function (38–44), governing the response of ccRCC to PT2399. Arguing against the former possibility, TKO cells, although sensitive to PT2399, were resistant to CDK4/6 inhibitors (Supplementary Fig. S12E and S12F). In support of a noncanonical cyclin D1 function, however, both WT cyclin D1 and the cyclin D1 K112E variant promoted PT2399 resistance in the TKO ccRCC cells, as measured by cell number (Fig. 5C–E; Supplementary Fig. S10C, S10D, S11C, and S11D) and G/1S progression (Supplementary Fig. S7C–S7F).

**Figure 6.**
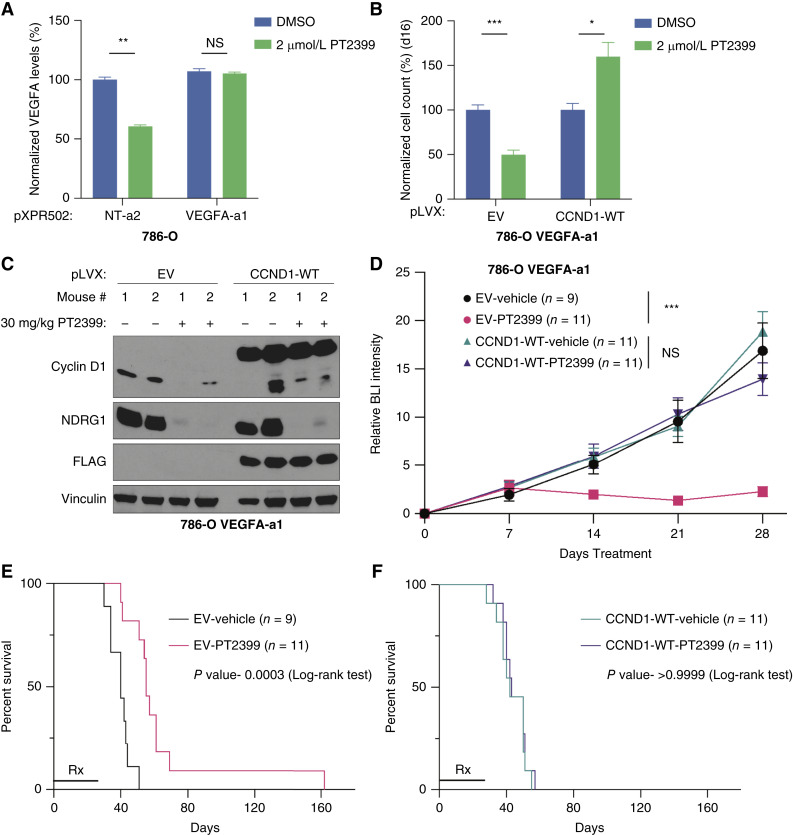
Failure to downregulate *VEGFA* and *CCND1* confers resistance to PT2399 *in vivo*. **A,** VEGFA levels from conditioned media of 786-O cells that stably express firefly luciferase, dCas9-VP64, and the indicated CRISPRa sgRNAs after treatment with 2 μmol/L PT2399 or DMSO for 48 hours. The VEGF levels were normalized to the NT-a2 expressing cells treated with DMSO. Data are the means ± SD of *n* = 3 biological replicates. **, *P* < 0.01, and NS, unpaired *t* test. **B,** Cellular proliferation assays of 786-O cells that stably express firefly luciferase, dCas9-VP64, CRISPRa sgRNA VEGFA-a1, and either cyclin D1 (WT) or the EV. The cells were treated with 2 μmol/L PT2399 or DMSO for 16 days. Data are the means ± SD of *n* = 3 biological replicates and were normalized to the DMSO-treated cells for the respective cell lines (EV or WT). *, *P* < 0.05; ***, *P* < 0.001; unpaired *t* test. **C,** Immunoblot analysis of orthotopic xenografts formed by cells from (**B**) that were treated with PT2399 (30 mg/kg) or vehicle daily for 5 days by oral gavage. **D,** Average relative BLI intensity over time of orthotopic xenografts formed by cells from (**B**) that were treated with PT2399 (30 mg/kg) or vehicle daily for 28 days by oral gavage. For each mouse, BLI readings were normalized to the BLI value from the day the treatment was started. Data are the mean ± SEM from three independent experiments. ***, *P* < 0.001, and NS, two-way ANOVA. **E** and **F,** Kaplan–Meier survival curves for mice in **D**. Rx bar indicates length of treatment. Log-rank (Mantel–Cox) test, with indicated *P* values. NS, not significant.

To ask whether sustained cyclin D1 activity could bypass the antiproliferative effects of PT2399 *in vivo*, we performed nude mouse xenografts assays with 786-O cells engineered to produce firefly luciferase. Exogenous expression of cyclin D1 using a constitutive promoter in these cells did not, by itself, prevent the antitumor effects of PT2399 (Supplementary Fig. S13A–S13D), likely due to the well-documented antitumor effects of downregulating the HIF2-responsive growth factor VEGF (5, 45). Next, we generated luciferase-positive 786-O cells that stably expressed VEGF using CRISPRa with a sgRNA that we empirically found to induce physiologically relevant VEGF levels (Fig. 6A; Supplementary Fig. S14A). These cells were then superinfected with lentiviruses expressing WT cyclin D1 or the EV and assayed for PT2399 sensitivity *in vitro* (Fig. 6B) or unilaterally injected into the kidneys of nude mice. Once the latter cells formed tumors, as determined by serial bioluminescence imaging (BLI), the mice were randomized to PT2399 or vehicle once daily by oral gavage. A subset of the mice were sacrificed for pharmacodynamic studies, which confirmed downregulation of NDRG1 and endogenous cyclin D1 for the mice treated with PT2399 (Fig. 6C). Sustained expression of cyclin D1 and VEGF, but not VEGF alone, prevented the antitumor effects of PT2399, as determined by serial BLI and mouse survival (Fig. 6D–F; Supplementary Fig. S14B and S14C).

**Figure 7.**
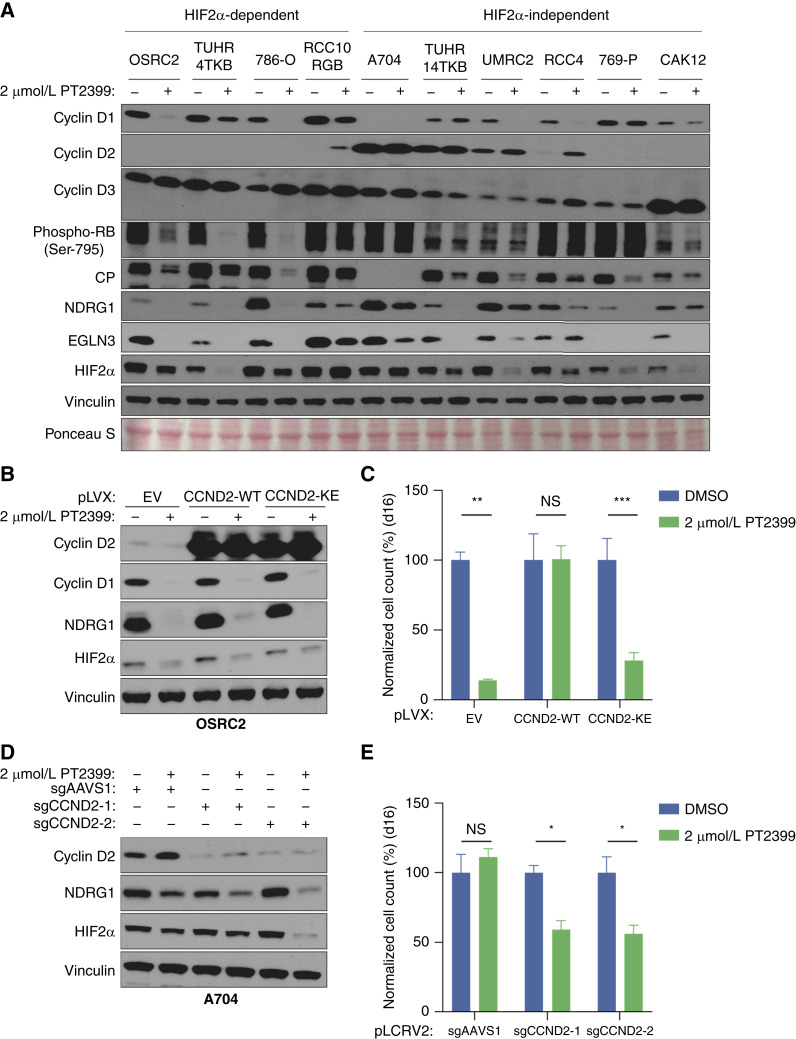
High cyclin D2 expression as a potential cause of *in vitro* HIF2 independence. **A,** Immunoblot analysis of indicated ccRCC cell lines that were treated with 2 μmol/L PT2399 or DMSO for 4 days. **B,** Immunoblot analysis of OSRC2 cells stably expressing cyclin D2 (WT or K111E) or the EV and treated with 2 μmol/L PT2399 or DMSO for 4 days. **C,** Cellular proliferation assays of cells as in (**B**) that were treated with 2 μmol/L PT2399 or DMSO for 16 days. Data are the means ± SD of *n* = 3 biological replicates and were normalized to the DMSO-treated cells for the respective cell lines (EV, WT, or KE). **, *P* < 0.01; ***, *P* < 0.001; NS, unpaired *t* test. **D,** Immunoblot analysis of A704 cells that underwent CRISPR editing with the indicated CRISPRko sgRNAs and were then treated with 2 μmol/L PT2399 or DMSO for 4 days. **E,** Cellular proliferation assays of cells as in (**D**) that were treated with 2 μmol/L PT2399 or DMSO for 16 days. Data are the means ± SD of *n* = 3 biological replicates and were normalized to the DMSO-treated cells for each respective sgRNA. *, *P* < 0.05, and NS, unpaired *t* test. NS, not significant; phospho = phosphorylated.

Many *VHL−/−* ccRCC cell lines are inexplicably HIF2-independent. Our prior surveys of HIF2-dependent and HIF2-independent *VHL−/−* ccRCC lines with respect to cell-cycle regulators did not yield an obvious explanation for cell-intrinsic HIF2 independence in this setting. Prompted by our cyclin D1 findings, we rephenotyped *VHL−/−* ccRCC lines and discovered that the proliferation of TUHR14TKB cells, although annotated to be HIF2-dependent (24), was unaffected by PT2399 or CRISPR-mediated elimination of HIF2α (Supplementary Fig. S15A–S15D). With this new categorization, we observed that multiple HIF2-independent *VHL−/−* ccRCC lines, including A704, TUHR14TKB, and UMRC2, expressed high levels of cyclin D2 (Fig. 7A). RCC4 cells induced cyclin D2 when treated with PT2399, and CAKI2 expressed cyclin D3 (Fig. 7A). We confirmed that exogenous expression of WT, but not KE, cyclin D2 was sufficient to confer resistance to PT2399 (Fig. 7B and C; Supplementary Fig S15E and S15F). Remarkably, CRISPR-mediated elimination of cyclin D2 in A704 partially sensitized them to PT2399 (Fig. 7D and E). These findings provide further evidence that the cell-intrinsic effects of HIF2 inhibitors in ccRCC are mechanistically linked to cyclin D1 downregulation.

## Discussion

We discovered that failure to downregulate cyclin D1 abrogates the antiproliferative effects of HIF2 inhibitors in ccRCC cells in cell culture and, in conjunction with maintenance of VEGF expression, in nude mice orthotopic xenografts assays and that the proproliferative effects of cyclin D1 require that it activate the CDK4 and/or CDK6 kinases. We also confirmed, by ChIP-seq and PRO-seq, that *CCND1* is a direct transcriptional target of HIF2 in ccRCC, as was suspected before (22, 30, 31, 46–49). Together with earlier studies showing that inhibition of cyclin D1 or CDK4/6 inhibits ccRCC *in vitro* and *in vivo* (45, 50–53), our work shows that downregulation of cyclin D1 is both necessary and sufficient for the suppression of cell-autonomous ccRCC proliferation by pVHL and by pharmacologic HIF2 inhibitors. Our studies illustrate the power of CRISPRa to probe drug mechanism of action, especially when the drug target is directly linked to transcription.

A polymorphism in a *CCND1* enhancer region that alters HIF2 binding has been linked to ccRCC risk, with increased HIF2 binding correlating with increased ccRCC risk (30–32). *CDKN2A/B*, which encodes CDK4/6 inhibitors p16 and p15, is inactivated by mutations or hypermethylation in a subset of ccRCC, and *CDKN2A/B* loss cooperates with *VHL* loss to promote ccRCC in mice (4, 26, 54, 55). These observations provide further genetic evidence for the importance of cyclin D1 in ccRCC.

We recently showed that cells lacking pVHL have an increased requirement for CDK4/6 kinase activity compared with their WT counterparts (50, 51). This differential sensitivity, however, seems to be HIF-independent and pRB-dependent, which we imagine is driven by a non-HIF substrate of pVHL (51). In contrast, our new findings describe an epistatic relationship between HIF2 and cyclin D1 that seems to be pRB-independent. The latter was surprising because the requirement for CDK4/6 activity in most cancers is strictly dependent on the canonical CDK4/6 substrate, pRB. Our studies suggest that the requirement for CDK4/6 kinase activity in ccRCC involves at least one CDK4/6 substrate beyond pRB, which could help explain the conspicuous absence of *RB1* mutations in ccRCC. p107, p130, or both, might be such substrates, especially as we observed enhanced binding of cyclin D1 to p107 in cells lacking pRB under substrate-trapping conditions.

We also discovered, however, that elimination of all three pRB family members in ccRCC cells did not completely negate the antiproliferative effects of PT2399 *in vitro* nor their reversal by cyclin D1. In this setting, in contrast to pRB family–proficient cells, cyclin D1’s ability to activate CDK4/6 was no longer strictly required. In the simplest view, induction of PT2399 resistance by cyclin D1 is caused by the phosphorylation of pRB family members and one or more of the previously described kinase-independent, prooncogenic, functions of cyclin D1 (38–44). CDK4/6 kinase activity would be required in cells retaining at least one pRB family member because the presence of an unphosphorylated pRB family member would impair cell-cycle progression despite these putative kinase-independent cyclin D1 activities. The latter, however, would be unmasked specifically in TKO cells (Fig. 5A–E; Supplementary Fig. S10A–S10D and S11A–S11D).

Our findings in three different ccRCC lines link HIF2 dependence to D-type cyclins. However, we purposely choose three cell lines that are particularly sensitive to HIF2α loss *in vitro* in hopes of increasing the magnitude of sgRNA enrichment in our CRISPRa screen. We do not yet know whether cyclin D1 is the critical target in all HIF2-dependent ccRCCs.

It will be important, moving forward, to ask whether cyclin D1 is a positive predictive biomarker for HIF2 inhibitors and whether dysregulation of cyclins, including cyclin D2, contribute to clinical resistance. A caveat is that in breast cancer preclinical models, in contrast to our ccRCC models, pRB inactivation is sufficient to cause resistance to CDK4/6 inhibitors (56). Even for breast cancer, however, dozens of resistance mechanisms have been described in patients who have become refractory to these agents, with pRB loss only accounting for about 5% of cases (57). Complicating things further, the antitumor effects of HIF2 inhibitors seems, based on prior reports and our current study, to involve both cell-intrinsic effects through cyclin D1 and cell-extrinsic effects through VEGF. In examining clinical samples obtained at the time of HIF2 inhibitor resistance, once such samples become available, it will also be important to remember that cyclin D1 is also a pharmacodynamic marker for HIF2 inhibitors. Thus, failure to downregulate cyclin D1 in such samples, absent examination of other HIF2 targets, could be a primary driver of resistance or merely a consequence of failure to engage HIF2.

Our search for functionally important HIF2 target genes was made possible by CRISPRa technology. Although our focus was on genes that, when reactivated, conferred resistance to HIF2 inhibition, we also identified genes that promoted or impaired ccRCC cell fitness irrespective of drug treatment. Among the former were c-*MYC* and SQSTM1, which are suspected of being the oncogenic targets on the ccRCC 8q and 5q amplicons [26, 27], respectively. Some of the genes that impaired ccRCC fitness when activated by CRISPRa map to chromosomal arms that are frequently deleted in ccRCC and hence are candidate haploinsufficient tumor-suppressor genes. More generally, CRISPRa should be a powerful technique for identifying the many genes that are suspected of contributing to cancer through copy number gains or losses.

Although CDK4/6 is believed to play a critical role in cancer proliferation, CDK4/6 inhibitors have very modest single-agent activity in cancer, possibly due to partial compensation by cyclin E/CDK2 in the absence of CDK4/6 (58–60). On the other hand, CDK4/6 inhibitors, including palbociclib, abemaciclib, and ribociclib, are now mainstays of hormone-responsive breast cancer treatment when combined with ER antagonists (60). In hormone-responsive breast cancer, ER drives the transcription of *CCND1* (61–65)*,* and thus combining a CDK4/6 inhibitor with an ER antagonist directly inhibits CDK4/6 catalytic activity and lowers cyclin D1 levels, respectively. Based on this logic, combining a HIF2 inhibitor with a CDK4/6 inhibitor should be additive or synergistic, at least in ccRCCs that are intrinsically HIF2-dependent, leading to deeper and more frequent tumor regressions. Our earlier preclinical studies testing such combinations are consistent with this view (51). The eventual cure for ccRCC, however, will also most certainly require combining treatments whose actions are orthogonal to the HIF2–cyclin D1 axis so as to minimize the emergence of resistance.

## Methods

### Cell Lines and Cell Culture

OSRC2 (RRID: CVCL_1626), TUHR4TKB (RRID: CVCL_5957), RCC10RGB (RRID: CVCL_1647), and TUHR14TKB (RRID: CVCL_5953) cells were obtained from RIKEN BioResource Research Center Cell Bank. 786-O (RRID: CVCL_1051), RCC4 (RRID: CVCL_0498), A704 (RRID: CVCL_1065), 769-P (RRID: CVCL_1050), CAKI2 (RRID: CVCL_0235), and 293FT (RRID: CVCL_6911) cells were obtained from the ATCC. UMRC2 cells were a gift of Bert Zbar and Marston Linehan, NCI, Bethesda. OSRC2, TUHR14TKB, and 769-P cells were grown in RPMI-1640 media (Gibco, 11875093). TUHR4TKB, 786-O, RCC10RGB, RCC4, UMRC2, and 293FT cells were grown in DMEM (Gibco, 11995065). A704 cells were grown in Eagle’s Minimum Essential Medium (Corning, MT10010CM). CAKI2 cells were grown in McCoy’s 5A media (Gibco, 16600108). All cell culture media were supplemented with 10% FBS (GeminiBio, 100-106) and 1% penicillin–streptomycin (Gibco, 15140122). Lentivirally infected cells were maintained in media supplemented with blasticidin (10 μg/mL), puromycin (2 μg/mL), or neomycin/G418 (1 mg/mL) based on the corresponding selectable markers.

Cells were cultured at 37°C in a humidified incubator containing 5% CO_2_. All cell lines were routinely verified to be *Mycoplasma*-negative using MycoAlert Mycoplasma Detection Kit (Lonza, LT07-318). The TUHR14TKB cell line was authenticated through short tandem repeat profile analysis using FTA Sample Collection Kit for Human Cell Authentication Service (ATCC, 135-XV), and the result of this analysis is listed in Supplementary Table S2.

### Chemicals

Palbociclib (Selleckchem, S1116) was prepared as a 10 mmol/L stock in DMSO and used at the indicated final concentration in the substrate-trapping and cell growth assays. Etoposide (Selleckchem, S1225) was prepared as a 10 mmol/L stock in DMSO and used at the indicated final concentration in the annexin V apoptosis assays. PT2399 was a gift from Merck & Co., Inc. For *in vitro* assays, PT2399 was prepared as a 10 mmol/L stock solution in DMSO. For *in vivo* assays, PT2399 was dissolved at a stock concentration of 7.5 mg/mL in a solution that contained 60% of 0.5% methyl cellulose (Fisher Scientific, M352-500) and Tween-80 (Sigma-Aldrich, P4780) in water, 30% poly(ethyleneglycol) 400 (Sigma-Aldrich, 202398), and 10% ethanol 200 proof (Fisher Scientific, 07-678-005).

### Plasmids

Lenti dCAS-VP64_Blast (Addgene #61425), the pXPR_502 CRISPRa sgRNA expression vector (Addgene # 96923), lentiCRISPR v2 CRISPRko sgRNA expression vector (Addgene # 52961), pLVX-2xFLAG-2xSTREP-CCND1-IRES-mCherry CCND1 expression vector (Addgene #172640), and pLVX-2xFLAG-2xSTREP-CCND2-IRES-mCherry CCND2 expression vector (Addgene #172627) were obtained from Addgene. The pLL3.7-EF1α-Fluc-Neo firefly luciferase expression vector was a gift from Matthew Oser, Dana-Farber Cancer Institute (DFCI).

To subclone either CRISPRa sgRNA sequences into pXRP_502 or CRISPRko sgRNA sequences into lentiCRISPR V2, corresponding vectors were digested and dephosphorylated at their cut ends by simultaneous incubation with FastDigest Esp3l (Thermo Fisher Scientific FD0454) and FastAP Thermosensitive Alkaline Phosphatase (Thermo Fisher Scientific EF0654) for 30 minutes at 37°C. The linearized vector was gel-purified using QIAquick Gel Extraction Kit (Qiagen, 28706). CRISPRa or CRISPRko sgRNA sequences were designed using CRISPick from the Broad Institute (https://portals.broadinstitute.org/gppx/crispick/public). The oligos for cloning sgRNA were purchased from Integrated DNA Technologies (IDT) and were dissolved in nuclease-free water at stock concentration of 100 μmol/L. To phosphorylate and anneal the oligonucleotides, sgRNA sense and antisense oligos were mixed in equimolar ratio in a reaction that contained T4 polynucleotide kinase (New England Biolabs, M0201L) and 10X T4 DNA ligation buffer with ATP (New England Biolabs, M0202L). The mixture was incubated for 30 minutes at 37°C and for 5 minutes at 95°C and cooled to 25°C at a rate of 5°C/minute in a thermocycler. The annealed oligos were diluted 1:100 in nuclease-free water. One μL of diluted oligos was then ligated with 50 ng of linearized pXPR-502 in a reaction containing 1 μL of 10X T4 DNA ligation buffer with 1 mmol/L ATP (New England Biolabs, M0202L) and 1 μL of T4 DNA ligase (New England Biolabs, M0202L), with final volume made up to 10 μL by nuclease-free water. The ligation mixture was incubated at room temperature (RT) for 4 hours. Then the ligation mixture was transformed into chemically competent HB101 *Escherichia coli* (Promega, L2011) and plated on ampicillin-containing LB-Agar plates, followed by overnight incubation at 30°C. Ampicillin-resistant single bacterial colonies were inoculated and expanded in ampicillin-containing liquid LB media. Plasmid DNA was extracted using QIAprep Spin Plasmid Miniprep Kit (Qiagen, 27106) and validated by Sanger sequencing. All the CRISPRa and CRISPRko sgRNA sequences used in this study are listed in Supplementary Table S3.

pLVX-2xFLAG-2xSTREP-CCND1 (K112E)-IRES-mCherry and pLVX-2xFLAG-2xSTREP-CCND2 (K111E)-IRES-mCherry were made by site-directed mutagenesis of corresponding vectors and oligos containing the desired mutation as listed in Supplementary Table S4 using Q5 Site-Directed Mutagenesis Kit (New England Biolabs, E0554S) as per the manufacturer’s instruction. The EV was made by excising the CCND1 cDNA from pLVX-2xFLAG-2xSTREP-CCND1 (K112E)-IRES-mCherry by inverse PCR and religation.

### HIF2α Subpool CRISPRa sgRNA Library

The HIF2α subpool CRISPRa sgRNA library was custom-synthesized and cloned into the pXPR_502 vector by the Genetic Perturbation Platform (Broad Institute). The HIF2α subpool sgRNA library targets 936 genes with ∼12 sgRNAs per gene. The 936 genes include (i) 294 genes whose CRISPRa guides were enriched in the PT2399-treated arm of the OSRC2 whole-genome CRISPRa screen (LFC > 0.5 and *P* < 0.05), (ii) 293 genes whose CRISPRa guides were depleted in the PT2399-treated arm of the OSRC2 whole-genome CRISPRa screen (LFC < -0.5 and *P* < 0.05), and (iii) 282 genes whose expression decreased after PT2399 in RNA-seq and PRO-seq datasets (LFC < −0.5 and *P* < 0.05). The library also targets other known HIF2 regulators (e.g., *HIF1*β (encoding ARNT), *EP300*, *TRAPP*, and *TRIM22*) and ccRCC oncogenes (e.g., *PAX8* and *SQSTM1*; See Supplementary Table S1). The library contains 300 nontargeting guides as controls and is available from the Broad Institute (CP1904).

### Immunoblot Analyses

Cell pellets were resuspended in RIPA buffer (Pierce, 89901) supplemented with a protease inhibitor cocktail (Roche, 11836170001) and phosphatase inhibitor cocktail (Roche, 4906845001). Cell lysates were then incubated on a rotator for 1 hour at 4°C. Cell lysates were clarified by centrifuging at 17000 × *g* in a centrifuge for 15 minutes at 4°C. The supernatant was transferred to a prechilled Eppendorf tube. Cell lysates were quantified using the Bradford protein assay (Bio-Rad, 5000006). Equal amounts of protein were mixed with Laemmli SDS-Sample Buffer (SB; 6X, reducing) to a final SB concentration of 1× and boiled at 95°C for 5 minutes. Protein lysates were loaded and resolved on 4% to 20% protein gels (Novex WedgeWell, XP04205BOX) and transferred onto 0.2 μmol/L nitrocellulose membranes using the Bio-Rad Trans-Blot Turbo Transfer System (Bio-Rad, 1704155). Membranes were briefly stained with Ponceau S (Cell Signaling Technology, 59803) to ensure equal loading and transfer. Membranes were then blocked with 5% nonfat milk in TBS with 0.1% Tween-20 (TBS-T) for a minimum of 1 hour and then probed with the indicated primary antibodies diluted in 5% BSA (GoldBio A-420-10) and 0.05% sodium azide in TBS-T overnight at 4°C. After overnight incubation, membranes were washed three times with TBS-T (10 minutes/wash). After washing, membranes were incubated with horseradish peroxidase–conjugated secondary antibodies that were diluted (1:5,000) in 5% nonfat milk in TBS-T for at least 30 minutes. After incubation, membranes were washed three times with TBS-T (10 minutes/wash). Bound antibodies were detected with pierce ECL Plus Western Blotting Substrate (Thermo Fisher Scientific, 32106), SuperSignal West Pico PLUS Chemiluminescent Substrate (Thermo Fisher Scientific, 34578), or Immobilon (Thermo Fisher Scientific, WBKLS0500). Chemiluminescent signal was detected with autoradiographic film (Denville, E3031). The autoradiographic films were then scanned with an HP scanner. The antibodies used in this study are listed in Supplementary Table S5.

### Lentivirus Production

A total of 1.4 × 10^6^ 293FT cells were seeded in 60-mm cell culture–treated dishes (Corning, 353002) in 3 mL of antibiotic-free DMEM supplemented with 10% FBS. The next morning, the cells were cotransfected with 1 μg of the desired lentiviral vector and the packaging helper plasmids psPAX2 (Addgene #12260; 0.75 μg) and pMD2.G (Addgene #12259; 0.25 μg) with Lipofectamine 2000 (Invitrogen, 11668019) according to the manufacturer’s instructions. Lentiviral particle–containing media were collected at 48 and 72 hours after transfection, pooled, filtered through 0.45-μmol/L SCFA filters (Corning, 431220), aliquoted, and stored at −80°C.

### Lentiviral Infection

Cells were seeded in 6-well plates (Fisher Scientific, 08-772-1B; 10^6^ cells in 1 mL of media per well). To each well, 1 mL of lentivirus particle–containing media was added, supplemented with 10 μg/mL polybrene (Millipore-Sigma, TR-1003-G). The 6-well plates were then spun at 90 × g for 2 hours at RT. Sixteen hours later, the virus-containing media were removed, and the cells were detached by a brief incubation with 0.25% trypsin-EDTA (Gibco, 25200072) at RT. The trypsin was then quenched with the fresh cell culture media containing 10% FBS. Infected cells were allowed to recover for a day and then selected with appropriate antibiotics: Blasticidin (10 μg/mL for OSRC2, 786-O, and TUHR4TKB), puromycin (2 μg/mL for OSRC2, 786-O, and TUHR4TKB), and neomycin/G418 (1,000 μg/mL for 786-O).

### CRISPRa Screen

For the whole-genome CRISPRa screens, 2.10 × 10^8^ OSRC2-dCas9-VP64 and TUHR4TKB-dCas9-VP64 cells were infected with Calabrese set A or B sgRNA library virus obtained from Genetic Perturbation Platform (Broad Institute; ref. 25). This number of cells was chosen to maintain a sgRNA representation at 1,000 cells/sgRNA assuming a multiplicity of infection (MOI) of 0.3. To do this, the cells were trypsinized, suspended, and mixed with library virus and 10 μg/mL of polybrene supplemented DMEM to achieve an MOI of 0.3. The cells (3 × 10^6^) were then plated into 2 mL/well aliquots in 6-well plates and centrifuged at 90 × *g* for 2 hours at RT. After overnight incubation, the virus-containing media were removed. The cells were then trypsinized, pooled, and replated in media appropriate for that cell line at a density of 3 × 10^6^ cells/150 mm tissue culture (TC)-treated cell culture dishes (Corning, 353025). Infected cells were allowed to recover for a day and then placed in fresh media supplemented with 2 μg/mL puromycin. After completion of puromycin selection, as determined by absence of any viable cells in uninfected control cells, the cells were detached using trypsin and split into plates with media containing DMSO or 2 μmol/L PT2399. On the same day (“d0”), two cell pellets (6 × 10^7^) were frozen for later extraction of genomic DNA. The remaining cells were passaged every fourth day, maintaining 6 × 10^7^ cells per treatment arm, and replated in fresh media containing either DMSO or 2 μmol/L PT2399. The screen was completed on d20. Genomic DNA was extracted from the d0 pellets and from similarly prepared d20 DMSO and d20 PT2399 cell pellets using QIAamp DNA Blood Maxi Kit (Qiagen, 51194). Genomic DNA was further purified using OneStep PCR Inhibitor Removal Kit (Zymo Research, D6030). DNA was then quantified using Qubit 1X dsDNA HS Assay Kit (Invitrogen, Q33231) on a Qubit 4 Flurometer (Invitrogen, Q33238). The purified genomic DNA was submitted to the Genetic Perturbation Platform (Broad Institute) for next-generation sequencing (NGS).

### HIF2α Subpool Library Screen

For the subpool screens, 4.8 × 10^7^ OSRC2-dCas9-VP64, TUHR4TKB-dCas9-VP64, and 786-O-dCas9-VP64 cells were infected with the HIF2α subpool CRISPRa sgRNA library at an MOI of 0.3, as discussed above. After completion of puromycin selection, cells were detached by using trypsin and split into plates with media either containing DMSO or 2 μmol/L PT2399 while maintaining 1.2 × 10^7^ cells per treatment arm. The remainder of the screen was done as described above for the whole-genome CRISPRa screen.

### Analysis of CRISPRa Screen

The reads from NGS were analyzed using the Apron tool from the Broad Institute for all cell lines and conditions. Differential analysis for sgRNA abundance was performed using Limma comparing replicates from pDNA, d0, and d20 time points. The mean sgRNA abundance of target gene was compared and plotted. The top 15 genes, sorted by effect size, were color-coded based on the average FC (log_2_; positive or negative). Genes with keywords multiple, potentially, LOC, LINC, inactive, no_site and one_intergenic_site were excluded from being labeled.

The statistical test to identify significance in CRISPRa datasets was a moderated *t* test provided by Limma package, which was further corrected using the Benjamini–Hochberg procedure.

### RNA-seq and Analysis

1 × 10^6^ cells/dish were plated in 100-mm TC-treated cell culture dishes (Corning, 353003) and treated with either DMSO or 2 μmol/L PT2399 in triplicate for 24 or 48 hours. The cells were then washed twice with ice-cold Dulbecco’s Phosphate Buffer Saline (DPBS; Gibco, 14190094) and scraped into 1 mL of ice-cold DPBS. The cells were pelleted by centrifugation at 1,200 × *g* for 5 minutes at 4°C. Cells pellets were flash frozen in the liquid nitrogen and stored at −80°C until ready for processing. RNA was extracted using Qiagen RNeasy Mini Kits (Qiagen, 74106). Total RNA was quantified and assessed for quality on a NanoDrop spectrophotometer (Thermo Fissher Scientific, ND-8000-GL). RNA samples were submitted to Genewiz for library construction and sequencing. ERCC RNA Spike-In Mix Kit (Thermo Fisher Scientific, 4456740) was added to normalize total RNA prior to library preparation following the manufacturer’s protocol. The RNA-seq libraries were prepared using NEBNext Ultra II RNA Library Prep Kit for Illumina (New England Biolabs, E7770L) according to the manufacturer’s instructions. Briefly, mRNAs were initially enriched with Oligod(T) beads. Enriched mRNAs were fragmented by incubating purified mRNA with NEBNext First-Strand Synthesis Reaction Buffer for 15 minutes at 94°C. First-strand and second-strand cDNA were subsequently synthesized. cDNA fragments were end-repaired and adenylated at 3′ends, and universal adapters were ligated to cDNA fragments, followed by index addition and library enrichment by PCR with limited cycles. The sequencing libraries were validated on an Agilent TapeStation (Agilent Technologies, G2991BA) and quantified using a Qubit 2.0 fluorometer and by qPCR (KAPA Biosystems, KK4824).

The sequencing libraries were clustered on lanes of a single flow cell. After clustering, the flow cell was loaded on the Illumina HiSeq instrument (4,000 or equivalent) according to the manufacturer’s instructions. The samples were sequenced using a 2 × 150 bp paired-end (PE) configuration. Image analysis and base calling were conducted by the HiSeq Control Software (HCS). Raw sequence data (.bcl files) generated from Illumina HiSeq was converted into fastq files and demultiplexed using Illumina’s bcl2fastq 2.17 software. One mismatch was allowed for index sequence identification.

PE fastq files were aligned to the hg38 genome using HISAT2. Gene-level read counts were quantified with featureCounts and normalized with DESeq2, fitting the average gene expression in each sample to a negative binomial distribution. Differential expression was assessed between PT2399- (24 or 48 hours) versus DMSO-treated samples using DESeq2.

Plots were generated for the differential comparisons between PT2399 (OSRC2 24 hours, OSRC2 48 hours, or TUHR4TKB 24 hours, TUHR4TKB 48 hours) versus DMSO. The top 15 genes, sorted by *P* value, were color-coded based on the average FC (log_2_; positive or negative). The statistical test to identify significance in RNA-seq datasets was a Wald test provided by the DESeq2 package, which was further corrected using the Benjamini–Hochberg procedure.

### Knock-In FLAG-HA Tag at the Endogenous *EPAS1* locus

Solid-phase reversible immobilization magnetic beads were prepared by washing 1 mL carboxylate-modified magnetic bead solution (GE Healthcare, 65152105050250) three times with 1 mL TE buffer (10 mmol/L Tris-HCl, pH 8.0, and 1 mmol/L EDTA, pH 8.0) using a magnetic stand (Invitrogen, 12321D). The beads were then resuspended in 50 mL DNA precipitation buffer [10 mmol/L Tris-HCl, pH 8.0, 1 mmol/L EDTA, pH 8.0, 1 mol/L NaCl, 18% w/v PEG-8000 (Sigma-Aldrich, 89510-250G-F), and 0.05% (v/v) Tween-20] and stored at 4°C. For each experiment, an aliquot of the beads was equilibrated to RT before use. A double-stranded homology-directed repair (HDR) DNA template was prepared using a previously published method (66) with modifications. A double-stranded DNA that was designed to introduce a 3X-FLAG-HA epitope tag and intervening linker to the N-terminus of *EPAS1* and which was flanked by 5′ and 3′ homology arms was synthesized by Genewiz and provided in a pUC–Genewiz–Amp plasmid. This plasmid was used as a template for high-output PCR amplification using Kapa HotStart polymerase (Roche, KK2601). To purify the resulting PCR product, 100 μL of the PCR reaction was mixed with 120 μL of SPRI magnetic beads in an Eppendorf tube, and the mixture was incubated for 10 minutes at RT. The Eppendorf tube was then placed on the magnetic stand for several minutes so that the beads would attach to the side of the Eppendorf tube. Next the liquid was removed, and the beads were washed twice with 1 mL of freshly prepared 70% ethanol while leaving the tube in the stand. After the final wash, the liquid was removed by gentle aspiration, and the beads were allowed to air-dry for 5 to 6 minutes. The Eppendorf tube was then removed from the magnetic stand, and the beads were resuspended in 12 μL of 2 mmol/L Tris-HCl, pH 8.0, and incubated for 10 minutes at RT. The Eppendorf tube was then placed back in the magnetic stand for several minutes to capture the beads, and the supernatant containing the purified HDR template was transferred to a new Eppendorf tube.

To produce Cas9–RNP complexes, 1.2 μL Alt-R CRISPR-Cas9 sgRNA [stock solution: 100 μmol/L in 10 mmol/L Tris-HCl, pH 8.0, and 1 mmol/L EDTA (TE) buffer, IDT], 1.7 μL of Alt-R S.p. Cas9 nuclease (stock solution: 62 μmol/L, IDT, 1081058), 1.4 μL of Alt-R Cas9 electroporation enhancer (stock solution: 100 μmol/L in TE buffer, IDT, 1075916), and 0.7 μL of DPBS was mixed and incubated for at least 10 minutes at RT. Meanwhile, 2 × 10^5^ cells were resuspended in 20 μL of SF-nucleofection buffer (Lonza Amaxa SF Cell Line 4D-Nucleofector X Kit, V4XC-2032) and added to the Cas9–RNP mixture together with 3 μg of HDR template (typically 1.5–2 μg/μL). This final mixture was transferred to Nucleocuvette strips (Lonza Amaxa SF Cell Line 4D-Nucleofector X Kit, V4XC-2032). The mixture was electroporated by using the 4D-Nucleofector X unit with the electroporation condition DS138 based on pilot experiments in which the electroporation efficiency for the cells to be electroporated was tested for the 15 manufacturer-provided electroporation conditions and manufacturer-provided GFP reporter plasmid. Then 100 μL of prewarmed media were added into each cuvette and incubated for 10 minutes at RT. Individual electroporation mixtures were transferred into individual wells of 6-well plates containing 2 mL/well of prewarmed media supplemented with 1 μmol/L of Alt-R HDR enhancer V2 (stock solution: 0.69 mmol/L in DMSO, IDT, 10007921). The media were removed 16 hours later and replaced with fresh media. Knock-in efficiency was determined by amplicon sequencing and immunoblot analysis 3 to 4 days after nucleofection. Single-cell cloning was conducted for OSRC2 cells with 3X-FLAG-HA knock-in at the N-terminal of the *EPAS1* locus and were used for the following ChIP-seq assays. The sequence of the Alt-R CRISPR-Cas9 sgRNA that targets the N-terminus of *EPAS1* and the sequence of the HDR template are listed in Supplementary Table S6.

### ChIP-seq

For ChIP sample preparation, 1 × 10^7^ adherent cells were washed with DPBS, trypsinized, transferred to Eppendorf tubes, and collected by centrifugation at 300 × *g* for 5 minutes at RT. Cell pellets were washed once with RT DPBS-complete [PBS supplemented with 1× protease inhibitor (Sigma, PIC0006) and 5 mmol/L sodium butyrate], resuspended in 2 mmol/L of disuccinimidyl glutarate (Thermo Fisher Scientific, 20593), diluted in PBS, and then agitated at 850 rpm on a Fisherbrand Isotemp Heat/Cool Programmable Thermal Mixer II (Thermo Fisher Scientific, 15600330) at RT for 45 minutes. The cell pellets were collected by centrifugation at 300 × *g* for 5 minutes at RT. The supernatant was removed, and the cell pellets were resuspended in freshly made 1% formaldehyde (Thermo Fisher Scientific, 28908) diluted in DPBS followed by agitation at 850 rpm on a Fisherbrand Isotemp Heat/Cool Programmable Thermal Mixer II for 10 minutes at RT. The cross-linking was quenched by adding glycine to a final concentration of 0.125 mol/L with agitation at 850 rpm on a Fisherbrand Isotemp Heat/Cool Programmable Thermal Mixer II for 5 minutes at RT. The cell pellets were collected by centrifugation at 300 × *g* for 5 minutes at 4°C. Cell pellets were washed once with ice-cold DPBS-complete. Cross-linked cells were then pelleted, snap-frozen, and stored at −80°C.

Aliquots of frozen pellets were thawed on ice, resuspended in 500 μL of 1% SDS lysis buffer (50 mmol/L Tris-HCl, pH 8.0, 10 mmol/L EDTA, pH 8.0, 1% SDS, 1× protease inhibitor, and 5 mmol/L sodium butyrate) and lysed on ice for 10 to 15 minutes. The lysates were transferred to 1 mL Adaptive Forced Acoustics fiber milliTUBEs (Covaris, 520135) and sonicated at the following settings: (140 peak incident power, 5% duty factor, and 200 cycles per burst) using a Covaris E220 sonicator. Sonication lasted for 2.5 minutes (per sample for ChIP-seq samples). After sonication, the lysates were transferred to Eppendorf tubes and cleared by centrifugation at 17,000 × *g* for 15 minutes at 4°C.

To determine the amount of precleared chromatin to be used for ChIP-seq experiments, 10 μL of the lysates were aliquoted (INPUT), and 90 μL of TE was added to make a 100 μL solution. One μL RNase A (Invitrogen, AM2696) was added to the samples and incubated for 30 minutes at 37°C, and then 5 μL Proteinase K (20 mg/mL, Thermo Fisher Scientific, EO0492) was directly added to the mixture and incubated at 65°C for 16 hrs with constant agitation at 850 rpm on a Fisherbrand Isotemp Heat/cool Programmable Thermal Mixer II at the indicated temperatures. Next, the chromatin was purified using PCR Purification Kit (Qiagen, 28104). Briefly, 500 μL PB buffer was added to each 100 μL sample, and the mixture was mixed and loaded onto the purification column. The purified chromatin was eluted using 32 μL EB buffer. Chromatin (INPUT) concentration was determined using Qubit High-Sensitivity Kit on a Qubit 4 Fluorometer, and 40 μg chromatin (INPUT) was immunoprecipitated with 5 μg of FLAG antibody (Sigma-Aldrich, F1804).

Antibody–bead conjugation was done by first taking 30 μL of protein G beads (Life Technologies, 10004D) per sample and washing them three times with 500 μL ice-cold 0.5% BSA diluted in DPBS. The beads were then resuspended in 500 μL ice-cold 0.5% BSA diluted in DPBS with the FLAG antibody and rotated end-to-end for 4 to 6 hours at 4°C, followed by two additional washes with ice-cold 0.5% BSA diluted in DPBS to remove unbound antibody. Lysates containing the desired amount of chromatin were diluted 10-fold using ChIP dilution buffer (20 mmol/L Tris-HCl, pH 8.0, 1% Triton X-100, 2 mmol/L EDTA, 150 mmol/L NaCl, 1× protease inhibitor, and 5 mmol/L sodium butyrate), added to prewashed antibody–bead conjugates, and then rotated end-to-end overnight at 4°C. The next day, the bead-bound chromatin–antibody complexes were washed twice with RIPA 0 buffer (10 mmol/L Tris-HCl, pH 7.4, 1 mmol/L EDTA, pH 8.0, 0.1% SDS, 1% Triton X-100, and 0.1% sodium deoxycholate), twice with RIPA 0.3 buffer (10 mmol/L Tris-HCl, pH 7.4, 1 mmol/L EDTA, pH 8.0, 0.1% SDS, 1% Triton X-100, 0.1% sodium deoxycholate, and 0.3 mol/L NaCl), and twice with LiCl buffer (10 mmol/L Tris-HCl, pH 7.4, 1 mmol/L EDTA, pH 8.0, 0.5% NP40, 0.5% sodium deoxycholate, and 0.25 mol/L LiCl) and then eluted with 100 μL prewarmed (37°C) ChIP elution buffer (1% SDS and 0.1 mol/L NaHCO_3_ in H_2_O). One μL RNase A was added to the samples and incubated for 30 minutes at 37°C, and then 5 μL proteinase K was added to the samples and incubated at 65°C for 16 hours. During the RNase A and proteinase K incubations, the samples were constantly agitated at 850 rpm on a Fisherbrand Isotemp Heat/cool Programmable Thermal Mixer II.

For ChIP-seq samples, the supernatant was purified using MinElute PCR Purification Kit (Qiagen, 28004), eluted in 12 μL H_2_O, and quantified using Qubit High-Sensitivity Kit. A 5 μL sample was used for ChIP-seq library preparation using Swift DNA Library Prep Kit (Swift Biosciences, 1002). 75 bp PE reads were sequenced on a NextSeq instrument (Illumina).

For peak calling and data analysis, all samples were processed through the computational pipeline developed at the DFCI Center for Functional Cancer Epigenetics primarily using open-source programs (67, 68). Sequence tags were aligned with Burrows–Wheeler Aligner (RRID: SCR_010910; ref. 69) to build hg19, and uniquely mapped, nonredundant reads were retained. These reads were used to generate binding sites with Model-Based Analysis of ChIP-seq 2 (MACS v2.1.1.20160309) with a *q* value (FDR) threshold of 0.01 (70). We evaluated multiple quality control criteria based on alignment information and peak quality: (i) sequence quality score, (ii) uniquely mappable reads (reads that could only map to one location in the genome), (iii) uniquely mappable locations (locations that could only be mapped by at least one read), (iv) peak overlap with Velcro regions, a comprehensive set of locations—also called consensus signal artifact regions—in the genome that have anomalous, unstructured high signal or read counts in NGS experiments independent of cell line and of experiment type, (v) number of total peaks (each sample had >3,000 peaks, passing the minimum requirement of 1,000), (vi) high-confidence peaks (the number of peaks that were enriched tenfold over the background), (vii) percentage overlap with known DHS sites derived from the ENCODE Project (samples meet the minimum 80% threshold), and (viii) peak conservation (a measure of sequence similarity across species based on the hypothesis that conserved sequences are more likely to be functional). All samples in this study passed quality control criteria.

To identify HIF2α peak location, the bed file was extended by 1, 5, and 10 kb for each peak region using BEDOPS tool (RRID: SCR_012865; ref. 71). The browser snapshot shown in Supplementary Fig. S4A was generated with bigWig files and Integrative Genomics Viewer (https://software.broadinstitute.org/software/igv/; RRID: SCR_011793).

### PRO-seq Sample Preparation and Analysis

A total of 3 × 10^6^ OSRC2 cells/dish were plated on d0 in triplicate in 150-mm TC-treated cell culture dishes. On day 3, the media were replaced with media containing PT2399 or DMSO, and the cells were harvested 6 hours later. The cells were detached by a brief incubation with 0.25% trypsin–EDTA (Gibco, 25200072) at 37°C. The trypsin was then quenched with ice-cold DMEM +10% FBS. The cells were then transferred to 50-mL conical tubes and kept on ice for the remainder of their processing.

Approximately 1 × 10^7^ cells per condition were collected by centrifugation at 300 × *g* for 4 minutes at 4°C. The supernatant was removed, and the cell pellets were gently resuspended in 10 mL ice-cold DPBS, followed by centrifugation at 300 × *g* for 4 minutes at 4°C. The supernatant was removed, and the cells were resuspended in 250 μL wash buffer [10 mmol/L Tris-HCl, pH 8.0, 10 mmol/L KCl, 250 mmol/L sucrose, 5 mmol/L MgCl_2_, 1 mmol/L EGTA, 10% (v/v) glycerol, 0.5 mmol/L DTT (Thermo Fisher Scientific, 707265ML), and 0.2 μL/mL RNase inhibitor (Invitrogen, AM2696) and protease inhibitor (Thermo Fisher Scientific, A32965)]. The cell pellets were gently pipetted using wide bore filtered pipette tips (Thermo Fisher Scientific, 2079GPK) to create single-cell suspensions. The cell suspension was passed through a cell strainer once (Falcon, 352235) to avoid cell clumps. Ten mL permeabilization buffer [10 mmol/L Tris-HCl, pH 8.0, 10 mmol/L KCl, 250 mmol/L sucrose, 5 mmol/L MgCl_2_, 1 mmol/L EGTA, 10% (v/v) glycerol, 0.1% (v/v) IGEPAL CA-630, 0.05% (v/v) Tween-20, 0.5 mmol/L DTT, and 0.2 μL/mL RNase inhibitor and protease inhibitor] was slowly (over 5–10 seconds) added to the single-cell suspension along the wall of the conical tube. The samples were then incubated on ice for 5 minutes before centrifugation at 400 × *g* for 4 minutes at 4°C. The supernatant was removed by gently inverting the tubes, after which 1 mL of ice-cold wash buffer was added to each of the pellets to dilute out the residual permeabilization buffer. The pellets were again gently pipetted two to three times to achieve a single-cell suspension. Next an additional 9 mL of ice-cold wash buffer was added to each tube, and the samples were pelleted by centrifugation at 400 × *g* for 4 minutes at 4°C. This wash procedure (1 mL wash buffer followed by 9 mL wash buffer) was repeated two more times. After the final wash, the cell pellets were resuspended in 250 μL freezing buffer [50 mmol/L Tris-HCl, pH 8.0, 40% (v/v) glycerol, 5 mmol/L MgCl_2_, 1.1 mmol/L EDTA, 0.5 mmol/L DTT, and 1 μL/mL RNase inhibitor] and transferred to 1.5-mL low-binding tubes (Thermo Fisher Scientific, Pl90410). Fresh 250 μL freezing buffer/tube was again added to the 50-mL conical tubes, followed by gentle pipetting, to collect any residual cells, which were then pooled with the first resuspension (bringing the total volume to 500 μL, which was further resuspended by gentle pipetting). Trypan blue–stained cell number was determined using a hemocytomer after which the cells were diluted using freezing buffer to the concentration of 10^6^ cell/100 μL. Aliquots of 5 × 10^6^ permeabilized cells per 1.5-mL low-binding tube were flash frozen in liquid nitrogen and stored at −80°C prior to use.

To conduct nuclear run-ons and library preparation, aliquots of frozen permeabilized cells were thawed on ice, pipetted gently, and recounted using a Luna II automated cell counter (Logos Biosystems). For each sample, 10^6^ permeabilized human cells were used for nuclear run-ons, with 5 × 10^4^ permeabilized *Drosophila* S2 cells added to each sample for normalization. Nuclear run-on assays and library preparation were performed as described in Reimer and colleagues (72) with modifications. Briefly, nascent RNA was labeled by a run-on assay in which an equal volume of 2X nuclear run-on buffer [10 mmol/L Tris-HCl, pH 8.0, 300 mmol/L KCl, 1% sarkosyl, 10 mmol/L MgCl_2_, 1 mmol/L DTT, 20 μmol/L/ea biotin-11-NTPs (Perkin Elmer, NEL543001EA), and 0.8 U/μL RNase inhibitor)] was added into permeabilized cell mixture and incubated for 5 minutes at 37°C. RNA was purified using Total RNA Purification Kit (Norgen Biotek Corp, 17200) per the manufacturer’s instructions, eluted in 100 μL RNAse-free water, and fragmented by base hydrolysis by adding 25 μL 5× fragmentation solution (375 mmol/L Tris-HCl, pH 8.3, 562.5 mmol/L KCl, and 22.5 mmol/L MgCl_2_) followed by incubation for 5 minutes at 94°C. Fragmentation was stopped by adding 125 μL ice-cold 0.1 mol/L EDTA. To enrich for biotin-labeled nascent RNA, 10 μL of streptavidin C1 magnetic beads (Invitrogen, 65001) were washed, rendered RNase-free per the manufacturer’s instructions, and resuspended in 50 μL binding buffer (10 mmol/L Tris-HCl, pH 7.4, 300 mmol/L NaCl, 0.1% Triton X-100) before being added to the fragmented RNA. The samples were rotated end-to-end for 20 minutes at RT. The streptavidin magnetic beads were then washed twice with high-salt buffer (50 mmol/L Tris-HCl, pH 7.4, 2 mol/L NaCl, and 0.5% Triton X-100), twice with binding buffer (listed above), and twice with low-salt buffer (5 mmol/L Tris-HCl, pH 7.4, and 0.1% Triton X-100). The beads were next resuspended in 500 μL TRIzol Reagent (Invitrogen, 15596026) and incubated at 65°C for 5 minutes to elute RNA from the beads. This elution step was repeated once, and the first and second eluates for a given sample were pooled. The RNA was subsequently purified by adding 2.75× volumes of 100% ethanol to the 400 μL solution recovered from aqueous phase of TRIzol in the presence of 20 μg of ultrapure glycogen (Invitrogen, 10814010). The pelleted RNA was subsequently washed per the manufacturer’s instructions and resuspended in 5 μL of 10 μmol/L App-VRA3_6N 3′ end adapter prepared using 5′ DNA Adenylation Kit (New England Biolabs, M2611A) and ligated using T4 RNA ligase 2, truncated KQ (New England Biolabs, M0373) per the manufacturer’s instructions with 15% (w/v) PEG-8000 in final concentration and incubated overnight at 16°C. 180 μL of betaine binding buffer [1.42 g of betaine (Sigma-Aldrich, 61962) in 10 mL with binding buffer] was mixed with ligations and incubated for 5 minutes at 65°C and then for 2 minutes on ice. Capture on streptavidin beads was again performed as above to enrich for ligated nascent RNAs except for the addition of a final wash with 1× MDE buffer (New England Biolabs, B0608S) after the washes with high-salt buffer, binding buffer, and low-salt buffer. To prepare nascent RNA for 5′ end adapter ligation, the 5′ ends of the RNA were first decapped by mRNA decapping enzyme (New England Biolabs, M0608S) for 1 hour at 37°C. The beads were washed once in high-salt buffer, once with low-salt buffer, and then once in 1X T4 PNK reaction buffer. The samples were then treated with T4 polynucleotide kinase (New England Biolabs, M0201) for 1 hour at 37°C for 5′-hydroxyl repairs, followed by one wash with high-salt buffer, one wash with low-salt buffer, and one wash with 0.25× T4 RNA ligase reaction buffer prior to resuspension in 5 μL of 10 pmol 5′ RNA adapter VRA5_6N. Next T4 RNA ligase 1 (New England Biolabs, M0204L) was used to ligate the 5′ RNA adapter per the manufacturer’s instructions with 15% (w/v) PEG-8000 in final solution and incubated overnight at 16°C. Following the 5′ end ligation, beads were washed twice each with high-salt buffer, binding buffer, and low-salt buffer and then once with 0.25× SuperScript IV reaction buffer. Reverse transcription was performed using SuperScript IV Reverse Transcriptase (Invitrogen, 18090010), and the cDNA was eluted from the beads by heating the samples twice for 30 seconds at 95°C. The eluted cDNA was initially amplified for five cycles of “preCR” [NEBNext Ultra II Q5 master mix (New England Biolabs, M0544S) with Illumina TruSeq PCR primers RP-1 and RPI-X for indexing] following the manufacturer’s suggested PCR cycling protocol for library construction. A portion of preCR was serially diluted and for test amplification to determine optimal amplification of final libraries. Libraries were amplified by three additional cycles of PCR (eight cycles in total). The amplified library was purified using the ProNex Size-Selective Purification System (Promega) and sequenced using the Illumina NovaSeq platform.

All the custom scripts used to process the PRO-seq data described herein are available on the AdelmanLab GitHub (https://github.com/AdelmanLab/NIH_scripts; 10.5281/zenodo.5519915). Dual, 6nt unique molecular identifiers (UMIs) were extracted from read pairs using UMI-tools (10.1101/gr.209601.116). Read pairs were trimmed using cutadapt 1.14. The UMI length was trimmed off the end of both reads to prevent read-through into the UMI of the other end of the sequence, which will happen for shorter fragments. An additional nucleotide was removed from the end of read 1 (R1), using seqtk trimfq (https://github.com/lh3/seqtk), to preserve a single-mate orientation during alignment. The PE reads were then mapped to a combined genome index, including both the spike (dm6, drosophila) and primary (hg19, human) genomes, using bowtie2 (10.1038/nmeth.1923). Properly paired reads were retained. These read pairs were then separated based on the genome (i.e., spike-in vs. primary) to which they mapped, and both these spike and primary reads were independently deduplicated, again using UMI-tools. Reads mapping to the reference genome were separated according to whether they were R1 or R2, sorted via samtools 1.3.1 (-n), and subsequently converted to bedGraph format using a custom script (bowtie2stdBedGraph.pl; 10.5281/zenodo.5519915). We note that this script counts each read once at the exact 3′ end of the nascent RNA. Because R1 in PRO-seq reveals the position of the RNA 3′ end, the “+” and “−” strands were swapped to generate bedGraphs representing 3′ end positions at a single-nucleotide resolution. Samples displayed highly comparable recovery of spike-in reads; thus, samples were normalized based on the DESeq2 size factors. Combined bedGraphs were generated by summing counts per nucleotide across replicates for each condition.

Annotated transcription start sites were obtained from human (GRCh37.87) Gene Transfer Formats from Ensembl. After removing transcripts with biotypes, PRO-seq signal in each sample was calculated in the window from the annotated transcription start site to +150 nt downstream, using a custom script, make_heatmap.pl. This script counts each read one time, at the exact 3′ end location of the nascent RNA. Given good agreement between replicates and similar return of spike-in reads, bedGraphs were merged within conditions and depth-normalized to generate bigWig files binned at 10 bp. The browser snapshot shown in Supplementary Fig. S4B was generated with bigWig files and Integrative Genomics Viewer (https://software.broadinstitute.org/software/igv/).

### Cell Proliferation Assay

Cells were seeded in 6-well plates (2 × 10^5^ cells in 2 mL of media per well). Two hours after plating of the cells, 1 mL of media containing DMSO or PT2399 (6 μM) was added to the well with the final concentration of 2 μmol/L PT2399. Cell growth assays were performed over 16 days. Cells were counted by ViCell XR (Beckman Coulter, 731050) on d4, d8, d12, and d16. After each count, the cells were replated back at a density of 2 × 10^5^ in fresh media containing DMSO or PT2399. The normalized cell counts on day 16 reported in the relevant figures represents the cumulative difference in cell growth between the conditions represented. This was calculated by multiplying the relative ratios between PT2399-treated cells and DMSO-treated cells at each of the four time points examined and normalizing the final result for each experimental condition as a percentage of the associated DMSO control, which was set to 100 percent. All proliferation assays were done at least as three independent biological replicates. Proliferation assays were similarly conducted for the cells nucleofected with RNPs containing Cas9 and sgAAVS1 or sgHIF2α.

### Nucleofection of Cas9-sgRNA RNP

For each Cas9-RNP reaction, 1.2 μL of the Alt-R CRISPR-Cas9 sgRNA [stock solution: 100 μmol/L in 10 mmol/L Tris-HCl, pH 8, and 1 mmol/L EDTA (TE) buffer, IDT], 1.7 μL of Alt-R S.p. Cas9 nuclease (stock solution: 62 μM, IDT, 1081058), 1.4 μL of Alt-R Cas9 electroporation enhancer (stock solution: 100 μmol/L in TE buffer, IDT, 1075916), and 0.7 μL of DPBS was mixed and incubated for at least 10 minutes at RT. Meanwhile, 2 × 10^5^ cells were resuspended in 20 μL of SF-nucleofection buffer (Lonza Amaxa SF Cell Line 4D-Nucleofector X Kit, V4XC-2032) and added to the 5 μL of Cas9–RNP mixture. This final mixture was transferred to Nucleocuvette strips (Lonza Amaxa SF Cell Line 4D-Nucleofector X Kit, V4XC-2032). The mixture was electroporated by using a 4D-Nucleofector X unit with the following electroporation conditions: DS138 for OSRC2, EH100 for TUHR4TKB, and EN138 for 786-O cells. A measure of 100 μL of prewarmed media were then added to each cuvette and incubated for 10 minutes at RT. Individual electroporation mixtures were transferred into individual wells of 6-well plates containing 2 mL/well of prewarmed media. For double or triple KO, 5 μL of Cas9–RNP mixture of individual sgRNA was constituted in the separate Eppendorf tube and mixed with the cells resuspended in the SF-nucleofection buffer (40 or 60 μL) right before the electroporation. The media were removed 16 hours later and replaced with fresh media. KO of the target genes was confirmed by immunoblot analysis 4 days after nucleofection. The Alt-R CRISPR-Cas9 sgRNAs used in this study are listed in Supplementary Table S7.

### Immunoprecipitation of Flag-Cyclin D1 under Substrate-Trapping Conditions

A total of 3 × 10^6^ OSRC2 cells expressing Flag-tagged cyclin D1 (WT or KE) or the EV were plated in 150-mm TC-treated cell culture dishes in 25 mL of RPMI, 10% FBS, and 1% PS media. On day 3, the media were replaced, and fresh media were added containing either DMSO or 2 μmol/L palbociclib. After 16 hours, the media were removed, and the cells were washed twice with ice-cold DPBS. The cells were then scraped in 2 mL PBS and transferred to prechilled 15-mL falcon tubes. Next the cells were pelleted by centrifuging at 1,200 × *g* for 5 minutes and flash-frozen by using liquid nitrogen and stored at −80°C freezer until further processing.

Frozen cell pellets were thawed on ice and were lysed in 50 mmol/L Tris-HCl, pH 7.4, 150 mmol/L NaCl, 1 mmol/L EDTA, and 1% Triton X-100 supplemented with protease inhibitor cocktail and phosphatase inhibitor cocktail (lysis/wash buffer). Cell lysates were then incubated on an end-to-end rotator for 1 hour at 4°C. Cell lysates were clarified by centrifuging at 17,000 × *g* for 15 minutes at 4°C. The supernatant was transferred in a prechilled Eppendorf tube. Cell lysates were quantified using the Bradford protein assay. One mg of protein lysate was mixed with 50 μL of Anti-FLAG M2 magnetic beads (Sigma-Aldrich, M8823) and then incubated on a rotator for 1 hour at 4°C. After 1 hour, the tubes were placed in a DynaMag-2 magnetic rack (Invitrogen, 12321D) to separate the magnetic beads with the bound proteins from the lysate. The beads were then washed five times with the wash buffer, after which bound proteins were eluted by incubation with 0.5 mg/mL FLAG peptide (Sigma-Aldrich, F4799) in a final volume of 100 μL of 50 mmol/L Tris-HCl, pH 7.4, and 150 mmol/L NaCl buffer for 30 minutes at 4°C. The FLAG-M2 magnetic beads were again separated using a DynaMag-2 magnetic rack, and the supernatants were transferred to prechilled Eppendorf tubes. The eluates were mixed with Laemmli SDS-SB (6×, reducing) to a final SB concentration of 1×, boiled at 95°C for 5 minutes, and immunoblotted as described above.

### Cell-Cycle Analysis

A total of 0.5 × 10^6^ cells/dish were plated in 100-mm TC-treated cell culture dishes and treated with either DMSO or 2 μmol/L PT2399 in triplicate for 4 days. The quantification of different cell-cycle stages of the treated cells was performed by using Cell Cycle Analysis Kit (Abcam, ab287852) according to the manufacturer’s instruction. Briefly, at the end of the treatment, cells were detached by a brief incubation with 0.25% trypsin–EDTA at RT. The trypsin was then quenched with the fresh cell culture media containing 10% FBS. The cells were pelleted by centrifuging at 400 × *g* for 5 minutes, and the supernatant was discarded. The cells were gently resuspended and then washed with 2 mL of ice-cold 1× cell-cycle analysis buffer and centrifuged at 400 × *g* for 5 minutes. The supernatant was then discarded, and the pelleted cells were fixed by adding 2 mL of 70% ethanol drop by drop while gently vortexing the cells. The cells were then incubated on ice for 30 minutes and then centrifuged at 400 × *g* for 5 minutes. The supernatant was then discarded, and the cells were washed one more time with 2 mL of ice-cold 1× cell-cycle analysis buffer, centrifuged, and stained with 500 μL of nuclear dye (propidium iodide) and enzyme A–containing staining solution for 30 minutes at RT. The quantification of stained cells was performed using the SRFortessa cell analyzer (BD Biosciences 649225) with BD FACSDiva software (RRID: SCR_001456), and the data were analyzed using FlowJo software (RRID: SCR_008520).

### Click-iT EdU Incorporation Assay

A total of 0.5 × 10^6^ cells/dish were plated in 100 mm TC-treated cell culture dishes and treated with either DMSO or 2 μmol/L PT2399 in triplicate for 4 days. The quantification of DNA synthesis in treated cells was performed using Click-iT Plus EdU Pacific Blue Flow Cytometry Assay Kit (Life Technologies, C10636) according to the manufacturer’s instruction. Briefly, at the end of the treatment, the cells were incubated with 10 μmol/L of EdU for 2 hours. The cells were then detached by a brief incubation with 0.25% trypsin–EDTA at RT, and trypsin was then quenched with the fresh cell culture media containing 10% FBS. The cells were pelleted by centrifuging at 400 × *g* for 5 minutes, after which the supernatant was discarded. The cells were then washed once with 3 mL of 1% BSA in DPBS and centrifuged at 400 × *g* for 5 minutes. The supernatant was discarded, and the pelleted cells were fixed by resuspension in 100 μL of Click-iT fixative. Following 15 minutes of incubation at RT, the cells were washed once with 3 mL of 1% BSA in DPBS and centrifuged at 400 × *g* for 5 minutes. The supernatant was discarded, and the pelleted cells were then permeabilized by resuspension in 100 μL of 1X Click-iT permeabilization and wash buffer. After the cells were incubated for 15 minutes at RT, 500 μL of Click-iT Plus reaction cocktail was added to the cells. The cells were incubated for an additional 30 minutes at RT while protecting the cells from light. The cells were then washed once with 3 mL of 1X Click-iT permeabilization and wash buffer and centrifuged at 400 × *g* for 5 minutes. The supernatant was discarded, and the pelleted cells were resuspended in 500 μL of 1X Click-iT permeabilization and wash buffer. The quantification of EdU was performed using the SRFortessa cell analyzer with BD FACSDiva software, and the data were analyzed using FlowJo software.

### Annexin V FITC Apoptosis Assay

A total of 0.5 × 10^6^ cells/dish were plated in 100-mm TC-treated cell culture dishes and treated with either DMSO or 2 μmol/L PT2399 or 2 μmol/L etoposide for 4 days in triplicate. The quantification of apoptosis in the treated cells was performed using Annexin V FITC Kit (BD Biosciences, BDB556420) according to the manufacturer’s instruction. Briefly, at the end of the treatment, cells were detached by a brief incubation with 0.25% trypsin–EDTA at RT. The trypsin was then quenched with the fresh cell culture media containing 10% FBS. The cells were pelleted by centrifugation at 400 × *g* for 5 minutes. The supernatant was discarded, and the cells were washed twice with 2 mL of ice-cold DPBS and centrifuged at 400 × *g* for 5 minutes. The supernatant was discarded, and the pelleted cells were resuspended in 100 μL of 1X annexin V binding buffer (BD Biosciences, BDB556454). The cells were then stained with 5 μL of annexin V FITC and incubated at RT for 15 minutes. The quantification of annexin V was performed using the SRFortessa cell analyzer with BD FACSDiva software, and the data were analyzed using FlowJo software.

### Analysis of DNA for Editing Efficiency

QIAamp DNA Blood Mini Kit (Qiagen, Catalog No. 51104) was used to extract genomic DNA from cell pellets of the OSRC2 or TUHR4TKB cells nucleofected with indicated sgRNAs from early passages (7 days) or late passages (30 days). Approximately 100 ng of genomic DNA from each cell line was then used in separate PCR reactions to generate ∼200 to 270 bp amplicons surrounding the site of the intended edit using oligos as listed in Supplementary Table S8. PCR was performed using KAPA HiFi HotStart ReadyMix (Roche Sequencing, 09420398001) with the following conditions: 3 minutes at 95°C, 30 cycles of 20 seconds at 98°C, 15 seconds at 72°C, and 15 seconds/kb at 72°C, followed by a final extension step for 5 minutes at 72°C. Amplicons were purified by using QIAquick PCR purification kit (Qiagen, 28106) and subjected to NGS (approximately at least 40,000 reads) through the Massachusetts General Hospital Center for Computational and Integrative Biology DNA Core. The editing efficiency was quantified by analysis of output fastq files using web-based CRISPResso2 program (http://crispresso2.pinellolab.org/; RRID: SCR_024503).

### Palbociclib Drug Curve

A total of 1,000 cells/well were seeded in a 96-well TC-treated cell culture plate (Corning, 0720090) and treated with DMSO or the indicated concentration of palbociclib for 8 days. On day 4, the media were replaced and fresh palbociclib was added. On day 8, the cells were washed once with 100 μL of DPBS and fixed by incubation for 10 minutes in 100 μL of 4% formaldehyde in PBS (Life Technologies, LC26754) at RT The fixed cells were then washed with 100 μL of DPBS and stained with 0.1% crystal violet (Sigma-Aldrich, C0775-25G) in 10% ethanol solution for 20 minutes. The crystal violet solution was aspirated from each well, and the adherent cells were washed with water three to four times to remove any excess stain solution. Stained cells were air-dried overnight and were destained by using 10% acetic acid, and absorbance of destained solution was measured at 590 nm. The palbociclib drug curves were plotted by normalizing absorbance values to DMSO control, which was set to 100 percent.

### ELISA and RT-qPCR for VEGFA

VEGF levels in conditioned media of cells treated with PT2399 or DMSO was measured in triplicate using Human VEGFA Quantikine ELISA Kit (R&D Systems, DVE00) according to the manufacturer’s instructions.

For RT-qPCR assay, after removal of the conditioned media, the cells were washed twice with ice-cold DPBS and scraped into Eppendorf tubes in 1 mL of ice-cold PBS. RNA was extracted and quantified as discussed earlier under RNA-seq. cDNA was reverse-transcribed from 1 μg purified RNA using AffinityScript qPCR cDNA Synthesis Kit (Agilent, 600559) with random primers and oligo-dT as the primer probes. cDNA for each sample was diluted tenfold for RT-qPCR. Multiplex TaqMan probe–based RT-qPCR was used, which allows mRNA quantification of the gene of interest (*VEGFA*) at the same time with a housekeeping gene (*ACTB*). Each RT-qPCR reaction consisted of 0.35 μL of FAM-conjugated VEGFA TaqMan probe (Applied Biosystems, 4351370 Hs00900055_m), 0.35 μL of a VIC-conjugated primer-limited β-actin TaqMan probe (Applied Biosystems, 4448485 Hs01060665_g), 1 μL of diluted cDNA, 2.8 μL of water, and 3.5 μL of TaqMan Multiplex Master Mix (Applied Biosystems, 4461882). RT-qPCR was performed in triplicate on a 384-well plate (Roche, 047297490001) on a LightCycler 480 Instrument II (Roche). All quantitative calculations were performed using the 2^−ΔΔCt^ method using *ACTB* as the reference gene. The Ct values for each probe were normalized to the Ct value of *ACTB* for that individual well. The data from each experiment were then normalized to the DMSO control to determine the relative FC in VEGFA mRNA expression.

### Generation of Cell Lines for Orthotopic Kidney Xenograft Assays

786-O cells were infected with lentivirus prepared from the pLL3.7-EF1α-Fluc-Neo vector to express firefly luciferase. Firefly luciferase activity of the successfully infected cells, as determined by neomycin resistance, was confirmed using the ONE-Glo Luciferase Assay System (Promega, E6120) as per the manufacturer’s instructions. (i) For the experiments in Supplementary Fig. S10, the 786-O-Fluc–expressing cells were infected with either EV-IRES-mCherry or CCND1-WT-IRES-mCherry viruses. Positive cells for mCherry expression were sorted using BD Aria II SORP at the DFCI Flow Cytometry Core. (ii) For the experiments in Fig. 7 and Supplementary Fig. S11, the 786-O Fluc–expressing cells were infected with lentivirus prepared from lenti dCAS-VP64_Blast to express dCas9-VP64 blast. After blasticidin selection, these cells were then infected with the lentiviruses expressing CRISPRa sgNT-a2 or sgVEGFA-a1 and placed under puromycin selection. Lastly, 786-O-Fluc-dCas9-VP64-sgVEGFA-a1 cells were infected with either EV-IRES-mCherry or CCND1-WT-IRES-mCherry viruses. Positive cells for mCherry expression were sorted using BD Aria II SORP at the DFCI Flow Cytometry Core. 786-O-Fluc-dCas9-VP64-sgVEGFA-a1-EV or 786-O-Fluc-dCas9-VP64-sgVEGFA-a1-CCND1-WT cells were used for *in vivo* assays.

### *In Vivo* Orthotopic Kidney Xenograft Assays

All animal experiments were carried out under DFCI protocol 04-019 that was approved by the Institutional Animal Care and Use Committee. NCRNU/F (Taconic) 6- to 8-week-old mice were anesthetized with subcutaneous injection of 120 mg/kg ketamine and 12 mg/kg xylazine cocktail from a stock solution containing a mix of 22 mg/mL ketamine and 0.2 mg/mL xylazine in 0.9% saline. An incision was made on the dorsal skin, and 1 × 10^6^ tumor cells in 20 µL DPBS were injected into the parenchyma of the visualized left kidney using a 27-gauge needle and 0.3-mL syringe. Mice received Ethiqa XR (3.25 mg/kg) as an analgesic agent during the recovery phase of the surgery. Tumors were monitored weekly by BLI beginning 2 weeks after surgery. For BLI imaging, mice were injected intraperitoneally with Rediject luciferin and anesthetized via inhalation of vaporized 99.9% liquid isoflurane (Covetrus, 029405). Sixty seconds later, the mice were imaged dorsally with auto exposure time from 1 to 180 seconds. BLI images were analyzed with Living Image Software 4.8.2 (PerkinElmer). Once the BLI signals increased for least two consecutive weeks, the mice were randomized (d0) to receive 30 mg/kg PT2399 or vehicle, via oral gavage, daily for 28 consecutive days. Mice were imaged weekly during the entire duration of the treatment regimen. Photon emission was normalized to the photon count on d0. The FC in photon count was calculated for the PT2399 or vehicle arms weekly. For survival analysis studies, the mice were sacrificed when they lost 20% of their body weight or when they seemed moribund or distressed. Two mice from each treatment arm were euthanized after 5 days of dosing for pharmacodynamic studies. The mice were euthanized and dissected, and tumors were extracted. Tumors lysates were prepared by sonicating (Fisher Scientific, FB50) tumor tissues in RIPA buffer supplemented with a protease inhibitor cocktail and phosphatase inhibitor cocktail. Cell lysates were then incubated on a rotator for 1 hour at 4°C. Cell lysates were clarified by centrifuging at 1,7000 × *g* in a centrifuge for 15 minutes at 4°C. Cell lysates were quantified using the Bradford protein assay. The lysates were mixed with Laemmli SDS-SB (6×, reducing) to a final SB concentration of 1×, boiled at 95°C for 5 minutes, and immunoblotted as described above.

### Statistical Analysis

Statistical analyses used for relevant experiments are indicated in the figure legends with relevant statistical tests used for the comparisons. Statistical analyses were performed using GraphPad Prism (RRID: SCR_002798) for all the experiments except CRISPRa, RNA-seq, and PRO-seq analyses.

### Data Availability

The RNA-seq, ChIP-seq, and PRO-seq raw and processed files were uploaded on Gene Expression Omnibus. All the datasets can be downloaded by using Gene Expression Omnibus accession number GSE277046 and are publicly available at https://www.ncbi.nlm.nih.gov/geo/query/acc.cgi?acc=GSE277046. The code used to analyze different datasets in this manuscript is publicly available at kaelinlabdfci GitHub (https://github.com/kaelinlabdfci/HIF2a_CCND1_ccRCC).

## Supplementary Material

Table S1 Table S1. Genes Targeted by sgRNAs in HIF2⍺ Subpool CRISPRa sgRNA Library (CP1904)

Table S2 Table S2. STR Profile Analysis of TUHR14TKB Cell Line

Table S3 Table S3. Sequences of Oligonucleotides Used for the Cloning of CRISPRa and CRISPRko sgRNAs

Table S4 Table S4. Sequences of Oligonucleotides Used to Modify pLVX Vector to Express Cyclin D1 K112E (KE), Cyclin D2 K111E (KE) Mutant, and Empty Vector (EV)

Table S5 Table S5. Antibodies Used in Immunoblotting Analyses

Table S6 Table S6. Sequence of the Alt-R CRISPR-Cas9 sgRNA That targets N-terminus of EPAS1 and the Sequence of the Homology Directed Repair Template

Table S7 Table S7. Sequences of Alt-R CRISPR-Cas9 sgRNAs

Table S8 Table S8. Sequences of Oligonucleotides Used to Determine the Efficiency of Cas9 Gene Editing at the RB1, RBL1, and RBL2 Loci

Shirole Fig. S1 Fig. S1: CRISPRa Screens for Modulators of ccRCC Sensitivity to HIF2alpha Inhibition

Shirole Fig. S2 Fig. S2: RNA-Seq Analysis of ccRCC Cells Treated with HIF2alpha Inhibitor PT2399

Shirole Fig. S3 Fig. S3: HIF2alpha Subpool CRISPRa Screen for Modulators of 786-O Cell Sensitivity to HIF2alpha Inhibition

Shirole Fig. S4 Fig. S4: CCND1 is a Direct Transcriptional Target of HIF2alpha

Shirole Fig. S5 Fig. S5: Failure to Downregulate CCND1 Confers Resistance to HIF2alpha Inhibition

Shirole Fig. S6 Fig. S6: Cyclin D1 Kinase Activity is Required for Cyclin D1 to Confer HIF2alpha-Independence

Shirole Fig. S7 Fig. S7: The HIF2 Inhibitor PT2399 Impairs G1/S Traversal by ccRCC Cells

Shirole Fig. S8 Fig. S8: HIF2alpha Inhibitor PT2399 Does Not Induce Apoptosis in ccRCC Cell Lines

Shirole Fig. S9 Fig. S9: Inactivation of pRB is Not Sufficient to Confer HIF2alpha-Independence

Shirole Fig. S10 Fig. S10: Inactivation of All 3 pRB Paralogs Does Not Fully Recapitulate Cyclin D1’s Ability to Confer HIF2alpha-Independence

Shirole Fig. S11 Fig. S11: Cyclin D1 Kinase Activity is Dispensable for Cyclin D1 to Confer HIF2alpha-Independence in the Cells Lacking All 3 pRB Paralogs

Shirole Fig. S12 Fig. S12: Loss of All 3 pRB Paralogs is Required to Confer Resistance to CDK4/6 Inhibitor

Shirole Fig. S13 Fig. S13: Sustained Expression of CCND1 Alone is Not Sufficient to Confer Resistance to PT2399 In Vivo

Shirole Fig. S14 Fig. S14: Failure to Downregulate VEGFA and CCND1 Confers Resistance to PT2399 In Vivo

Shirole Fig. S15 Fig. S15: High Cyclin D2 Expression as a Potential Cause of In Vitro HIF2-independence
