# Supplementary material for: Requirement for Cyclin D1 Underlies Cell-Autonomous HIF2 Dependence in Kidney Cancer
Source: Cancer Discov. 2025 Apr 4;15(7):1484–504. doi: 10.1158/2159-8290.CD-24-1378 (PMC12223508; doi:10.1158/2159-8290.CD-24-1378)
Supplement: Shirole Fig. S1 — Fig. S1: CRISPRa Screens for Modulators of ccRCC Sensitivity to HIF2alpha Inhibition [file cd-24-1378_shirole_fig.s1_suppsf1.pdf]

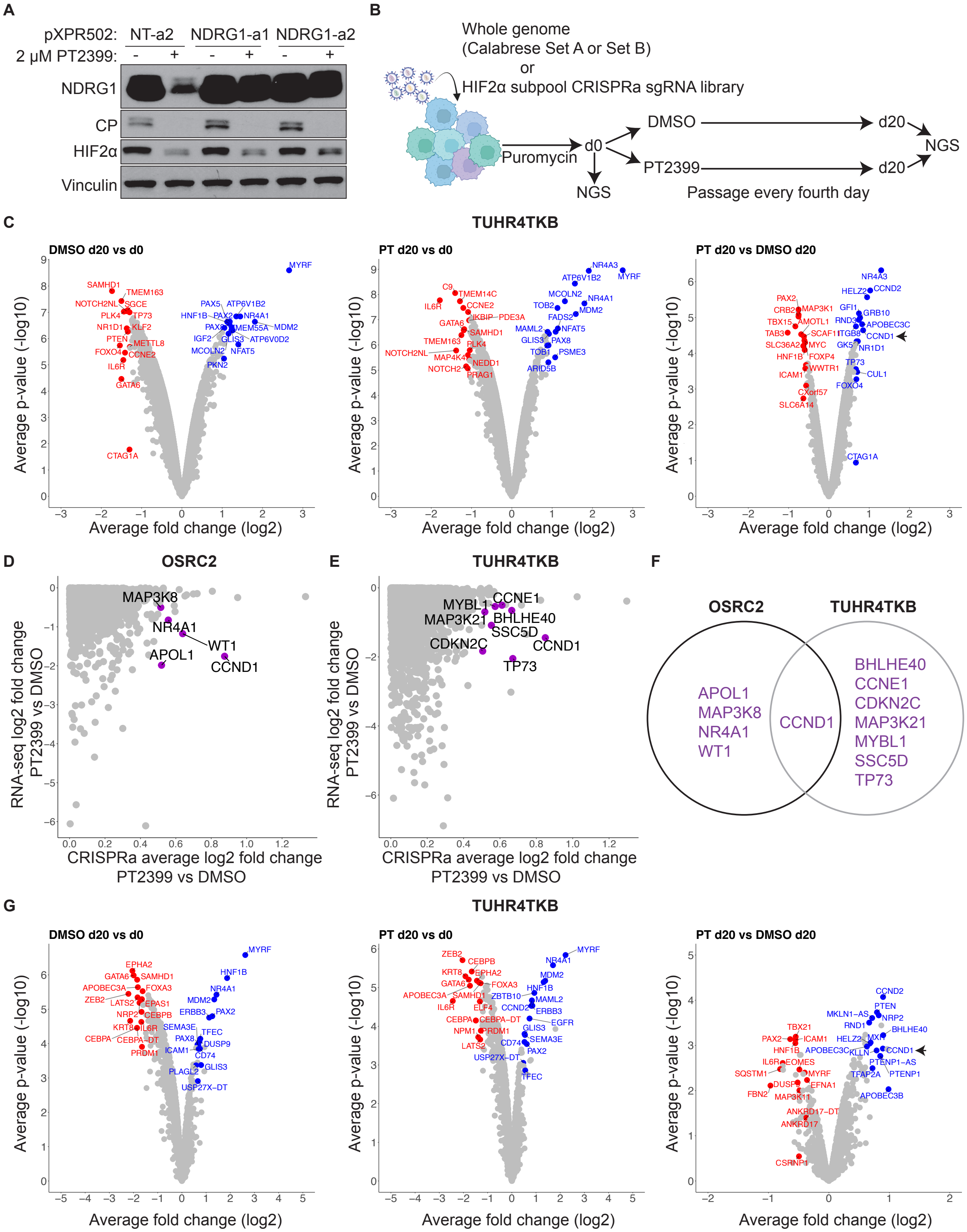
A
pXPR502:
NT-a2
NDRG1-a1
NDRG1-a2
2 µM PT2399:
- + - + - +
NDRG1
CP
HIF2α
Vinculin
B
Whole genome
(Calabrese Set A or Set B)
or
HIF2α subpool CRISPRa sgRNA library
Puromycin
d0
NGS
DMSO
PT2399
d20
d20
NGS
Passage every fourth day
C
TUHR4TKB
DMSO d20 vs d0
PT d20 vs d0
PT d20 vs DMSO d20
Average p-value (-log10)
Average fold change (log2)
MYRF
SAMHD1
TMEM163
NOTCH2NL
SGCE
PLK4
TP73
KLF2
NR1D1
PTEN
METTL8
FOXO4
CCNE2
IL6R
GATA6
CTAG1A
PAX5
ATP6V1B2
HNF1B
PAX2
NR4A1
PAX8
TMEM55A
MDM2
IGF2
GLIS3
ATP6V0D2
MCOLN2
NFAT5
PKN2
NR4A3
ATP6V1B2
MCOLN2
NR4A1
TOB2
MDM2
C9
TMEM14C
IL6R
CCNE2
GATA6
IKBIP
PDE3A
FADS2
MAML2
NFAT5
GLIS3
PAX8
TMEM163
PLK4
TOB1
PSME3
NOTCH2NL
MAP4K4
NEDD1
ARID5B
NOTCH2
PRAG1
NR4A3
HELZ2
CCND2
PAX2
CRB2
MAP3K1
GFI1
GRB10
TBX15
AMOTL1
RND3
APOBEC3C
TAB3
SCAF11
ITGB8
CCND1
SLC36A2
MYC
GK5
NR1D1
HNF1B
FOXP4
TP73
WWTR1
CUL1
ICAM1
FOXO4
CXorf57
SLC6A14
CTAG1A
D
OSRC2
RNA-seq log2 fold change PT2399 vs DMSO
CRISPRa average log2 fold change PT2399 vs DMSO
MAP3K8
NR4A1
WT1
APOL1
CCND1
E
TUHR4TKB
MYBL1
CCNE1
MAP3K21
BHLHE40
SSC5D
CDKN2C
CCND1
TP73
F
OSRC2
TUHR4TKB
APOL1
MAP3K8
NR4A1
WT1
CCND1
BHLHE40
CCNE1
CDKN2C
MAP3K21
MYBL1
SSC5D
TP73
G
TUHR4TKB
DMSO d20 vs d0
PT d20 vs d0
PT d20 vs DMSO d20
Average p-value (-log10)
Average fold change (log2)
MYRF
EPHA2
HNF1B
GATA6
SAMHD1
APOBEC3A
FOXA3
NR4A1
ZEB2
LATS2
EPAS1
MDM2
ERBB3
PAX2
NRP2
CEBPB
SEMA3E
KRT8
IL6R
TFEC
CEBPA
CEBPA-DT
PAX8
DUSP9
PRDM1
ICAM1
CD74
PLAGL2
GLIS3
USP27X-DT
ZEB2
CEBPB
NR4A1
KRT8
EPHA2
MDM2
GATA6
FOXA3
HNF1B
APOBEC3A
SAMHD1
ZBTB10
MAML2
IL6R
ELF4
CCND2
ERBB3
CEBPA
CEBPA-DT
EGFR
NPM1
PRDM1
GLIS3
LATS2
CD74
SEMA3E
PAX2
USP27X-DT
TFEC
CCND2
PTEN
MKLN1-AS
NRP2
RND1
BHLHE40
TBX21
PAX2
ICAM1
HELZ2
MX1
HNF1B
APOBEC3C
KLLN
CCND1
IL6R
EOMES
PTENP1-AS
SQSTM1
MYRF
TFAP2A
PTENP1
DUSP1
EFNA1
FBN2
MAP3K11
APOBEC3B
ANKRD17-DT
ANKRD17
CSRNP1

Fig. S1: CRISPRa Screens for Modulators of ccRCC Sensitivity to HIF2α Inhibition

A, Immunoblot analysis of TUHR4TKB cells expressing dCas9-VP64 that were infected with indicated CRISPRa sgRNAs and treated with 2 μM of the HIF2α inhibitor PT2399 or DMSO for 4 days. **B,** Schematic of CRISPR activation (CRISPRa) screens performed in HIF2α-dependent ccRCC cells that were either infected with whole genome or HIF2α subpool (see text) CRISPRa sgRNA libraries in the presence or absence of the 2 μM PT2399. **C,** Volcano plots showing genes whose sgRNAs were enriched or depleted in DMSO d20 vs d0 (left), PT2399 (PT) d20 vs d0 (middle), and PT d20 vs DMSO d20 (right) in TUHR4TKB cells expressing dCas9-VP64 that were infected with whole genome CRISPRa library. The top 15 genes based on average fold change (log 2) whose sgRNAs were enriched (blue) or depleted (red) are labeled. Arrowheads indicate the location of *CCND1* on the volcano plots. n = 2 biological replicates. **D,** Scatterplots of genes whose sgRNAs were enriched in PT2399 vs DMSO arm of CRISPRa screens (x-axis) in the OSRC2 cells compared to genes whose expression was downregulated by treatment with PT2399 for 48 hrs (y-axis). Genes with CRISPRa average log2 fold change > 0.5 and p-value < 0.05 and RNA-Seq average log2 fold change < 0.5 and p-value < 0.05 were labeled and colored purple. **E,** Scatterplots of genes whose sgRNAs were enriched in PT2399 vs DMSO arm of CRISPRa screens (x-axis) in the TUHR4TKB cells compared to genes whose expression was downregulated by treatment with PT2399 for 48 hrs (y-axis). Genes with CRISPRa average log2 fold change > 0.5 and p-value < 0.05 and RNA-Seq average log2 fold change < 0.5 and p-value < 0.05 were labeled and colored purple. **F,** Venn diagram of showing overlap between the genes from OSRC2 (**D**) and TUHR4TKB (**E)** cells. **G,** Volcano plots showing genes whose sgRNAs were enriched or depleted in DMSO d20 vs d0 (left), PT2399 (PT) d20 vs d0 (middle), and PT d20 vs DMSO d20 (right) in TUHR4TKB cells expressing dCas9-VP64 that were infected with HIF2α subpool CRISPRa library. The top 15 genes based on average fold change (log 2) whose sgRNAs were enriched (blue) or depleted (red) are labeled. Arrowheads indicate the location of *CCND1* on the volcano plots. n = 2 biological replicates.
