# Supplementary material for: Requirement for Cyclin D1 Underlies Cell-Autonomous HIF2 Dependence in Kidney Cancer
Source: Cancer Discov. 2025 Apr 4;15(7):1484–504. doi: 10.1158/2159-8290.CD-24-1378 (PMC12223508; doi:10.1158/2159-8290.CD-24-1378)
Supplement: Shirole Fig. S2 — Fig. S2: RNA-Seq Analysis of ccRCC Cells Treated with HIF2alpha Inhibitor PT2399 [file cd-24-1378_shirole_fig.s2_suppsf2.pdf]

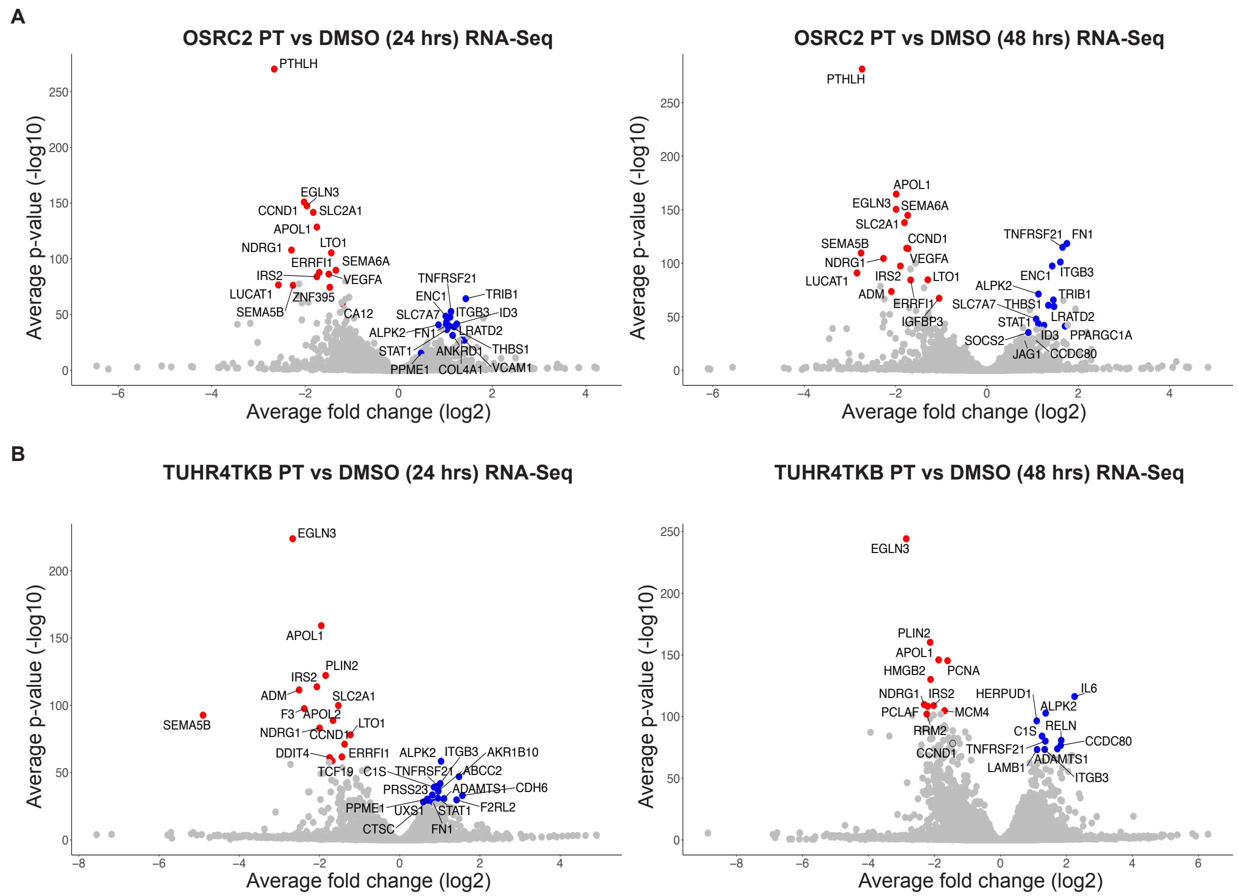

Fig. S2: RNA-Seq Analysis of ccRCC Cells Treated with HIF2α Inhibitor PT2399

A and B, Volcano plot showing genes whose expression were downregulated or upregulated in OSRC2 (**A**) and TUHR4TKB (**B**) cells after the treatment with 2 μM PT2339 for 24 hrs (left) or 48 hrs (right), as determined by RNA-Seq.
