# Supplementary material for: Requirement for Cyclin D1 Underlies Cell-Autonomous HIF2 Dependence in Kidney Cancer
Source: Cancer Discov. 2025 Apr 4;15(7):1484–504. doi: 10.1158/2159-8290.CD-24-1378 (PMC12223508; doi:10.1158/2159-8290.CD-24-1378)
Supplement: Shirole Fig. S3 — Fig. S3: HIF2alpha Subpool CRISPRa Screen for Modulators of 786-O Cell Sensitivity to HIF2alpha Inhibition [file cd-24-1378_shirole_fig.s3_suppsf3.pdf]

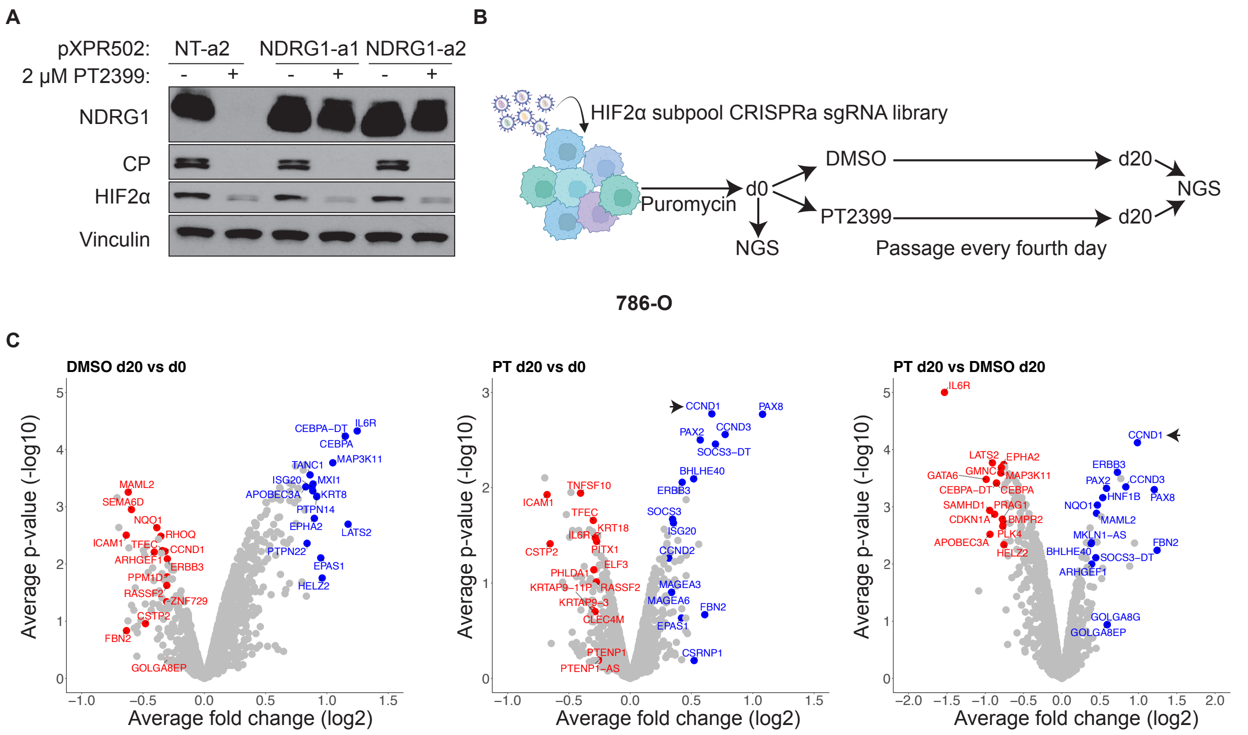

Fig. S3: HIF2α Subpool CRISPRa Screen for Modulators of 786-O Cell Sensitivity to HIF2α Inhibition

A, Immunoblot analysis of 786-O cells expressing dCas9-VP64 that were infected with indicated CRISPRa sgRNAs and treated with 2 μM PT2399 or DMSO for 4 days. **B,** Schematic of CRISPR activation (CRISPRa) screen performed in 786-O cells expressing dCas9-VP64 that were infected with HIF2α subpool CRISPRa sgRNA library in the presence or absence of 2 μM PT2399. **C,** Volcano plot showing genes whose sgRNA were enriched or depleted in DMSO d20 vs d0 (left), PT2399 (PT) d20 vs d0 (middle), and PT vs DMSO d20 (right) in 786-O cells expressing dCas9-VP64 that were infected with HIF2α subpool CRISPRa sgRNA library. The top 15 genes based on average fold change (log 2) whose sgRNAs were enriched (blue) or depleted (red) are labeled. Arrowheads indicate the location of *CCND1* on the volcano plots. n = 2 biological replicates.
