# Supplementary material for: Requirement for Cyclin D1 Underlies Cell-Autonomous HIF2 Dependence in Kidney Cancer
Source: Cancer Discov. 2025 Apr 4;15(7):1484–504. doi: 10.1158/2159-8290.CD-24-1378 (PMC12223508; doi:10.1158/2159-8290.CD-24-1378)
Supplement: Shirole Fig. S4 — Fig. S4: CCND1 is a Direct Transcriptional Target of HIF2alpha [file cd-24-1378_shirole_fig.s4_suppsf4.pdf]

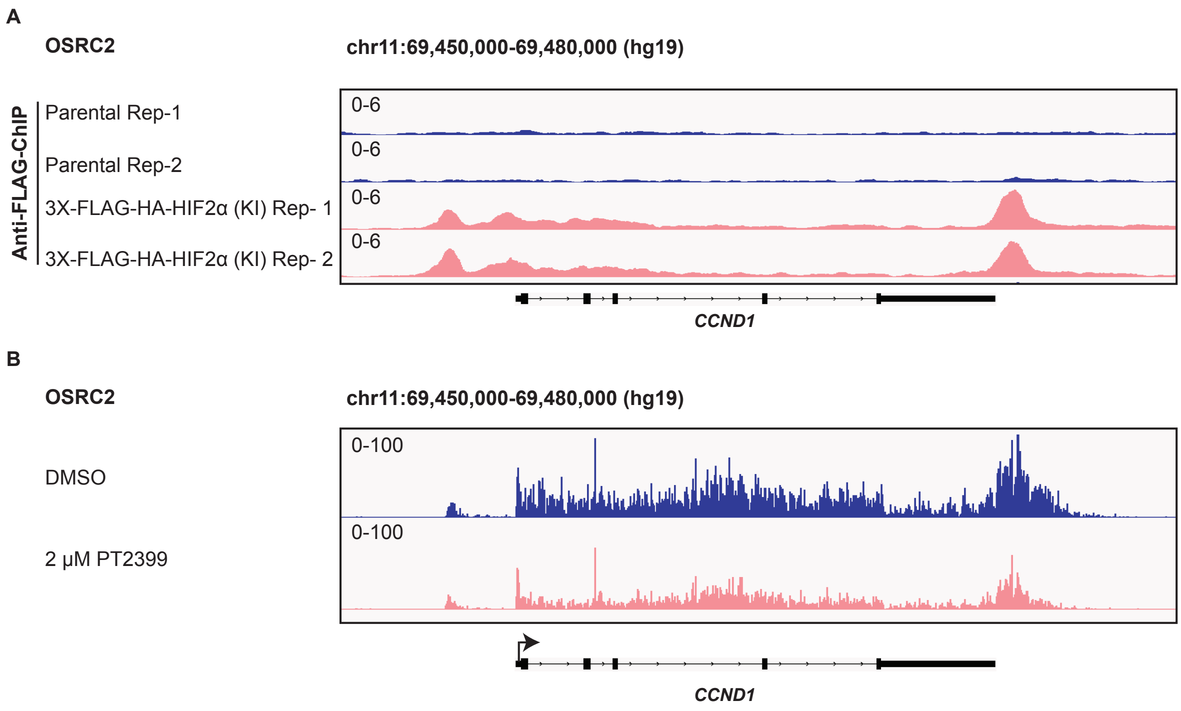

Fig. S4: *CCND1* is a Direct Transcriptional Target of HIF2α

A, Anti-FLAG ChIP-Seq tracks at the *CCND1* genomic locus of OSRC2 cells in which a 3XFLAG-HA epitope tag coding sequence was inserted at the 5' end of the endogenous *EPAS1* open reading frame by CRISPR-HDR based knock-in (KI) (Red) compared to Parental cells without KI (Blue). **B,** Nascent RNA tracks of *CCND1* in OSRC2 cells that were treated with 2 μM PT2399 or DMSO for 6 hrs. Also shown is the direction of the transcription for the *CCND1* genomic locus.
