# Supplementary material for: Requirement for Cyclin D1 Underlies Cell-Autonomous HIF2 Dependence in Kidney Cancer
Source: Cancer Discov. 2025 Apr 4;15(7):1484–504. doi: 10.1158/2159-8290.CD-24-1378 (PMC12223508; doi:10.1158/2159-8290.CD-24-1378)
Supplement: Shirole Fig. S5 — Fig. S5: Failure to Downregulate CCND1 Confers Resistance to HIF2alpha Inhibition [file cd-24-1378_shirole_fig.s5_suppsf5.pdf]

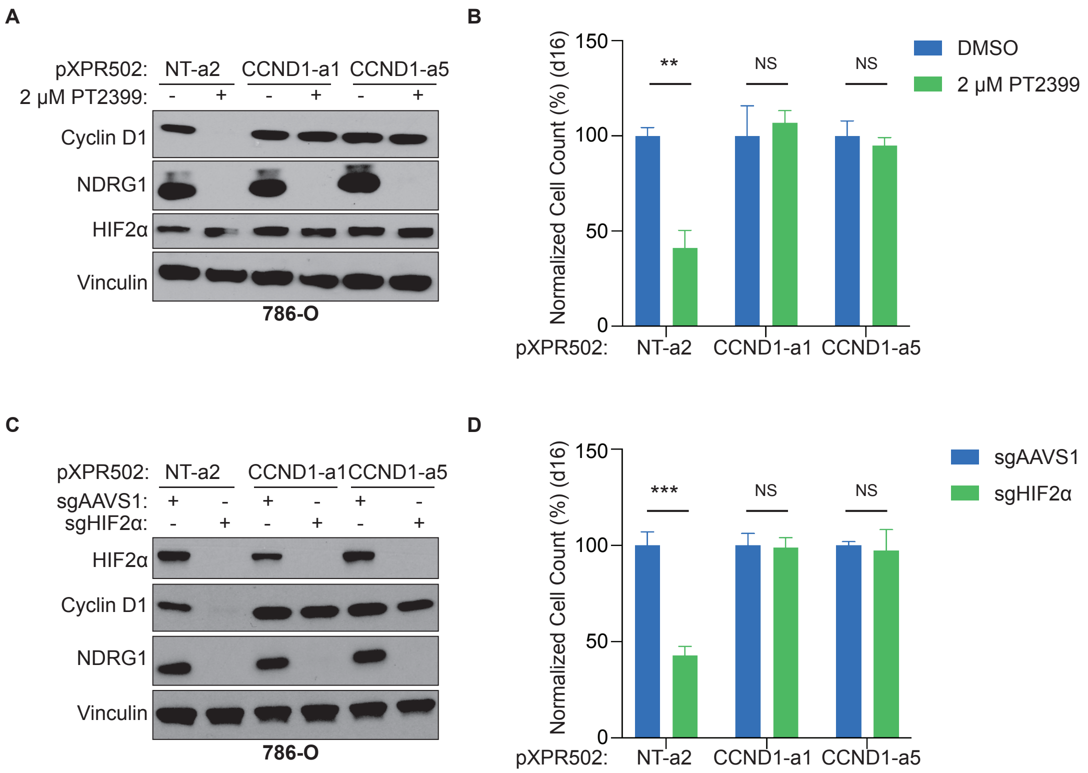

Fig. S5: Failure to Downregulate *CCND1* Confers Resistance to HIF2α Inhibition

A, Immunoblot analysis of 786-O cells expressing dCas9-VP64 that were infected with indicated CRISPRa sgRNAs and treated with 2 μM PT2399 or DMSO for 4 days. **B,** Cellular proliferation assays of cells as in (**A**) that were treated with 2 μM PT2399 or DMSO for 16 days. Data are means ± SD of n = 3 biological replicates and were normalized to the DMSO-treated cells for each respective sgRNA. **, P < 0.001 and NS (not significant), Unpaired t test. **C,** Immunoblot analysis of 786-O cells expressing dCas9-VP64 that were infected with indicated CRISPRa sgRNAs and subsequently nucleofected with RNPs containing Cas9 and either sgAAVS1 or sgHIF2α. **D,** Cellular proliferation assays of cells as in (**C**). Data are means ± SD of n = 3 biological replicates and were normalized to the sgAAVS1 cells for each of the respective CRISPRa sgRNAs. ***, P < 0.001 and NS, Unpaired t test.
