# Supplementary material for: Requirement for Cyclin D1 Underlies Cell-Autonomous HIF2 Dependence in Kidney Cancer
Source: Cancer Discov. 2025 Apr 4;15(7):1484–504. doi: 10.1158/2159-8290.CD-24-1378 (PMC12223508; doi:10.1158/2159-8290.CD-24-1378)
Supplement: Shirole Fig. S6 — Fig. S6: Cyclin D1 Kinase Activity is Required for Cyclin D1 to Confer HIF2alpha-Independence [file cd-24-1378_shirole_fig.s6_suppsf6.pdf]

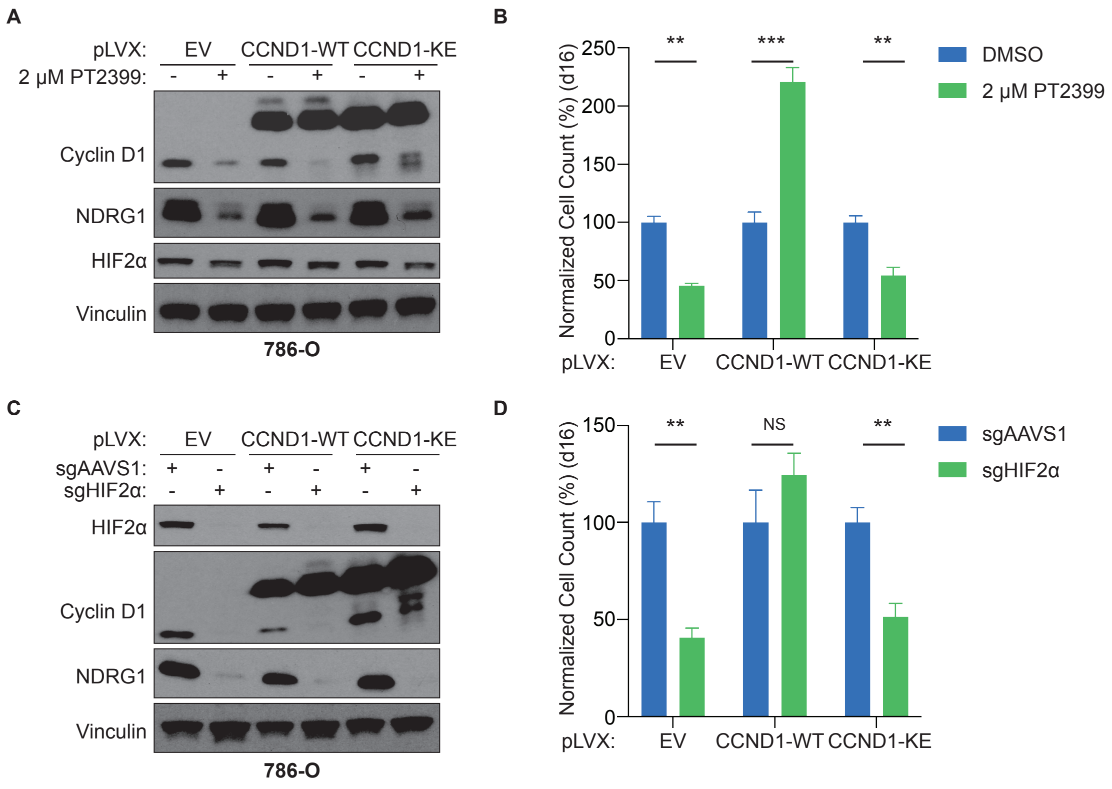

Fig. S6: Cyclin D1 Kinase Activity is Required for Cyclin D1 to Confer HIF2α-Independence

A, Immunoblot analysis of 786-O cells stably expressing Cyclin D1 (wild-type or K112E) or the empty vector (EV) and treated with 2 μM PT2399 or DMSO for 4 days. **B,** Cellular proliferation assays of cells as in (**A**) that were treated 2 μM PT2399 or DMSO for 16 days. Data are means ± SD of n = 3 biological replicates and were normalized to the DMSO-treated cells for the respective cell lines (EV, WT, or KE). **, $P < 0.01$, ***, $P < 0.001$, and NS, Unpaired t test. **C,** Immunoblot analysis of 786-O cells stably expressing Cyclin D1 (wild-type or K112E) or the empty vector (EV) and subsequently nucleofected with RNPs containing Cas9 and either sgAAVS1 or sgHIF2α. **D,** Cellular proliferation assays of cells as in (**C**). Data are means ± SD of n = 3 biological replicates and were normalized to the sgAAVS1 cells for the respective cell lines (EV, WT, or KE). **, $P < 0.01$ and NS, Unpaired t test.
