# Supplementary material for: Requirement for Cyclin D1 Underlies Cell-Autonomous HIF2 Dependence in Kidney Cancer
Source: Cancer Discov. 2025 Apr 4;15(7):1484–504. doi: 10.1158/2159-8290.CD-24-1378 (PMC12223508; doi:10.1158/2159-8290.CD-24-1378)
Supplement: Shirole Fig. S7 — Fig. S7: The HIF2 Inhibitor PT2399 Impairs G1/S Traversal by ccRCC Cells [file cd-24-1378_shirole_fig.s7_suppsf7.pdf]

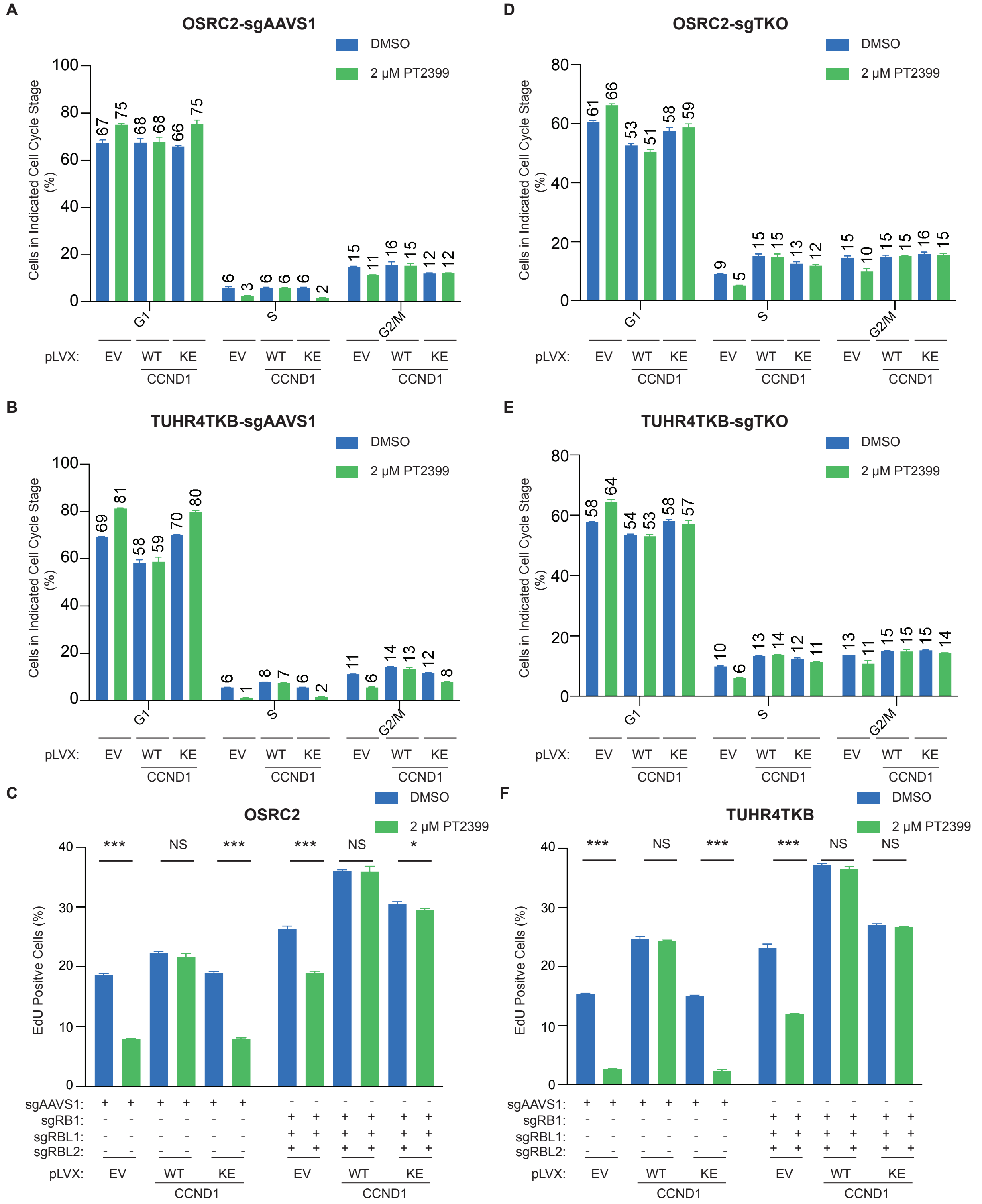

Fig. S7: The HIF2 Inhibitor PT2399 Impairs G1/S Traversal by ccRCC Cells

A and B, Cell cycle analysis of the indicated cells that were treated with 2 μM PT2399 or DMSO for 4 days. The number on the top of each bar indicates the percentage of the

cells present in the corresponding stages of the cell cycle. **C**, EdU incorporation was measured by FACS for OSRC2 cells stably expressing Cyclin D1 (wild-type or K112E) or the empty vector (EV) that were nucleofected with RNPs containing Cas9 and the indicated sgRNAs and then treated with 2 μM PT2399 or DMSO for 4 days. Data are means ± SD of n = 3 biological replicates. *, $P < 0.05$, ***, $P < 0.001$, and NS, Unpaired t test. **D and E,** Cell cycle analysis of the indicated cells that were treated with 2 μM PT2399 or DMSO for 4 days. The number on the top of each bar indicates the percentage of the cells present in the corresponding stages of the cell cycle. **F**, EdU incorporation was measured by FACS for TUHR4TKB cells stably expressing Cyclin D1 (wild-type or K112E) or the empty vector (EV) that were nucleofected with RNPs containing Cas9 and the indicated sgRNAs and then treated with 2 μM PT2399 or DMSO for 4 days. Data are means ± SD of n = 3 biological replicates, ***, $P < 0.001$ and NS, Unpaired t test.
