# Supplementary material for: Requirement for Cyclin D1 Underlies Cell-Autonomous HIF2 Dependence in Kidney Cancer
Source: Cancer Discov. 2025 Apr 4;15(7):1484–504. doi: 10.1158/2159-8290.CD-24-1378 (PMC12223508; doi:10.1158/2159-8290.CD-24-1378)
Supplement: Shirole Fig. S8 — Fig. S8: HIF2alpha Inhibitor PT2399 Does Not Induce Apoptosis in ccRCC Cell Lines [file cd-24-1378_shirole_fig.s8_suppsf8.pdf]

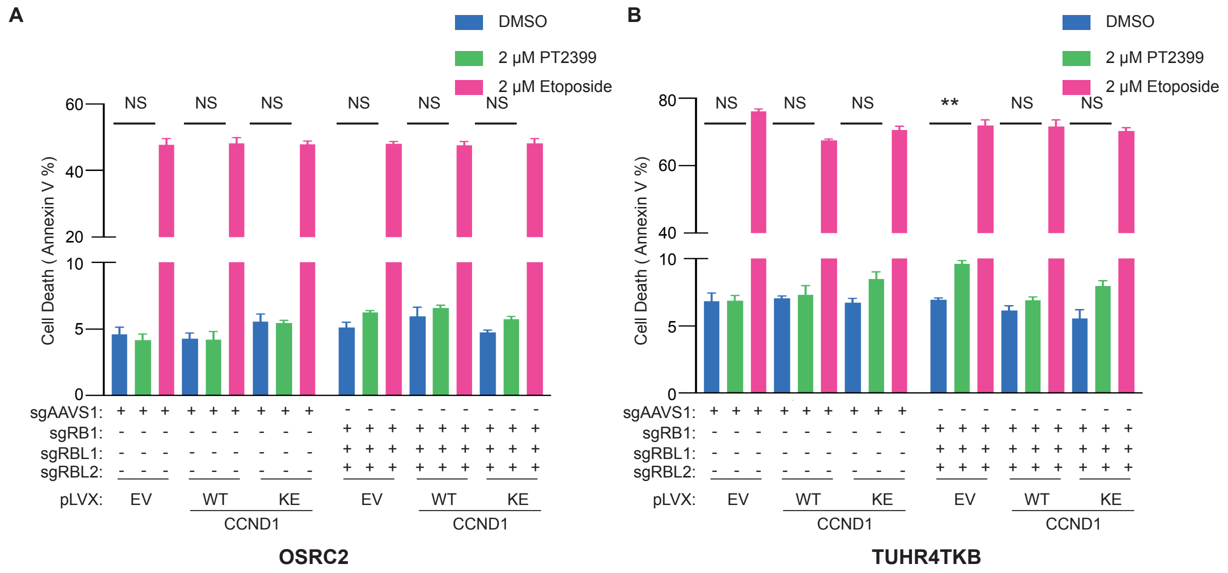

Fig. S8: HIF2α Inhibitor PT2399 Does Not Induce Apoptosis in ccRCC Cell Lines

A and B, Cell death was measured by FACS after Annexin V staining of OSRC2 (**A**) and TUHR4TKB (**B**) cells stably expressing Cyclin D1 (wild-type or K112E) or the empty vector (EV) that were nucleofected with RNPs containing Cas9 and the indicated sgRNAs and then treated with 2 μM PT2399 or 2 μM Etoposide or DMSO for 4 days. Data are means ± SD of n = 3 biological replicates. **, $P < 0.01$ and NS, Unpaired t test.
