# Supplementary material for: Requirement for Cyclin D1 Underlies Cell-Autonomous HIF2 Dependence in Kidney Cancer
Source: Cancer Discov. 2025 Apr 4;15(7):1484–504. doi: 10.1158/2159-8290.CD-24-1378 (PMC12223508; doi:10.1158/2159-8290.CD-24-1378)
Supplement: Shirole Fig. S10 — Fig. S10: Inactivation of All 3 pRB Paralogs Does Not Fully Recapitulate Cyclin D1’s Ability to Confer HIF2alpha-Independence [file cd-24-1378_shirole_fig.s10_suppsf10.pdf]

A

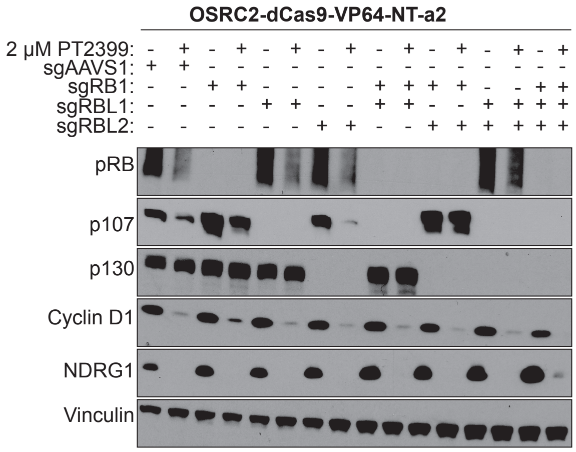
OSRC2-dCas9-VP64-NT-a2
2 µM PT2399: - + - + - + - + - + - + - + - +
sgAAVS1: + + - - - - - - - - - - - - - -
sgRB1: - - + + - - - - + + + + - - + +
sgRBL1: - - - - + + - - + + - - + + + +
sgRBL2: - - - - - - + + - - + + + + + +
pRB
p107
p130
Cyclin D1
NDRG1
Vinculin

B

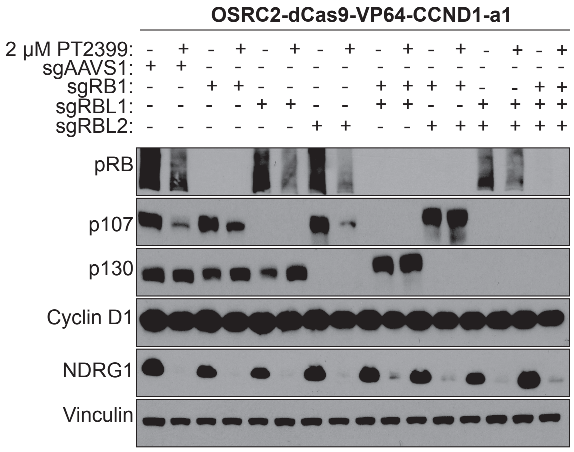
OSRC2-dCas9-VP64-CCND1-a1
2 µM PT2399: - + - + - + - + - + - + - + - +
sgAAVS1: + + - - - - - - - - - - - - - -
sgRB1: - - + + - - - - + + + + - - + +
sgRBL1: - - - - + + - - + + - - + + + +
sgRBL2: - - - - - - + + - - + + + + + +
pRB
p107
p130
Cyclin D1
NDRG1
Vinculin

C

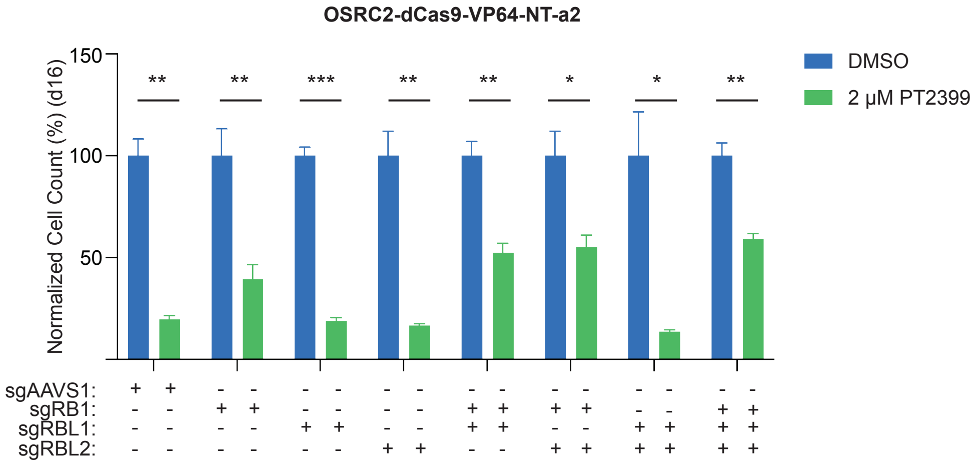
OSRC2-dCas9-VP64-NT-a2
Normalized Cell Count (%) (d16)
150
100
50
** ** *** ** ** * * **
DMSO
2 µM PT2399
sgAAVS1: + + - - - - - - - - - - - - - -
sgRB1: - - + + - - - - + + + + - - + +
sgRBL1: - - - - + + - - + + - - + + + +
sgRBL2: - - - - - - + + - - + + + + + +

D

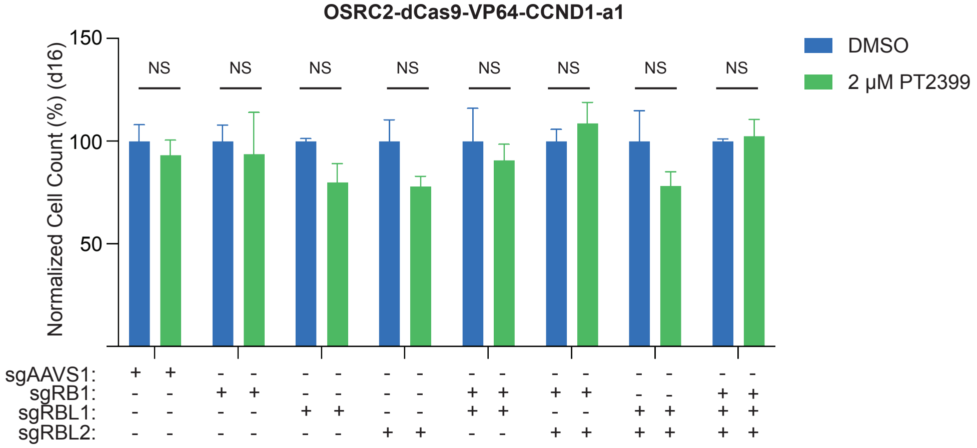
OSRC2-dCas9-VP64-CCND1-a1
Normalized Cell Count (%) (d16)
150
100
50
NS NS NS NS NS NS NS NS
DMSO
2 µM PT2399
sgAAVS1: + + - - - - - - - - - - - - - -
sgRB1: - - + + - - - - + + + + - - + +
sgRBL1: - - - - + + - - + + - - + + + +
sgRBL2: - - - - - - + + - - + + + + + +

Fig. S10: Inactivation of All 3 pRB Paralogs Does Not Fully Recapitulate Cyclin D1's Ability to Confer HIF2α-Independence

A and B, Immunoblot analysis of OSRC2 cells expressing dCas9-VP64 that were infected with CRISPRa sgRNAs NT-a2 **(A)** or CCND1-a1 **(B)**, nucleofected with RNPs containing Cas9 and the indicated sgRNAs, and then treated with 2 μM PT2399 or DMSO for 4 days. **C and D,** Cellular proliferation assays of cells as in (**A**) that were treated with 2 μM PT2399 or DMSO for 16 days. Data are means ± SD of n = 3 biological replicates and were normalized to the DMSO-treated cells for the respective combination of sgRNAs. *, $P < 0.05$, **, $P < 0.01$, and ***, $P < 0.001$, Unpaired t test. **D,** Cellular proliferation assays of cells as in (**B**) that were treated with 2 μM PT2399 or DMSO for 16 days. Data are means ± SD of n = 3 biological replicates and were normalized to the DMSO-treated cells for the respective combination of sgRNAs. NS, Unpaired t test.
