# Supplementary material for: Requirement for Cyclin D1 Underlies Cell-Autonomous HIF2 Dependence in Kidney Cancer
Source: Cancer Discov. 2025 Apr 4;15(7):1484–504. doi: 10.1158/2159-8290.CD-24-1378 (PMC12223508; doi:10.1158/2159-8290.CD-24-1378)
Supplement: Shirole Fig. S11 — Fig. S11: Cyclin D1 Kinase Activity is Dispensable for Cyclin D1 to Confer HIF2alpha-Independence in the Cells Lacking All 3 pRB Paralogs [file cd-24-1378_shirole_fig.s11_suppsf11.pdf]

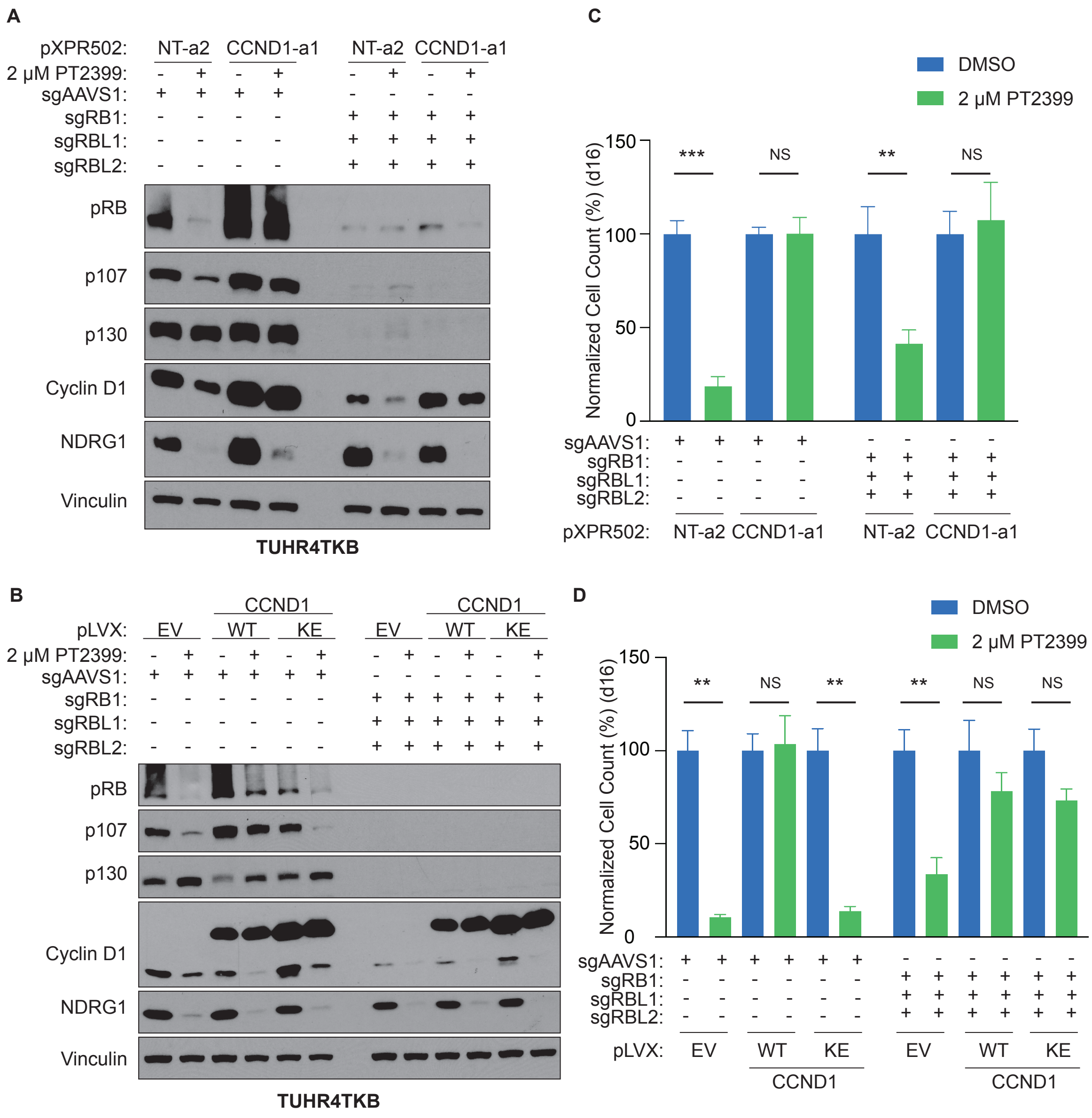

Fig. S11: Cyclin D1 Kinase Activity is Dispensable for Cyclin D1 to Confer HIF2α-Independence in the Cells Lacking All 3 pRB Paralogs

A, Immunoblot analysis of TUHR4TKB cells expressing dCas9-VP64 that were infected with indicated CRISPRa sgRNAs, nucleofected with RNPs containing Cas9 and the indicated sgRNAs, and then treated with 2 µM PT2399 or DMSO for 4 days. **B,** Immunoblot analysis of TUHR4TKB cells stably expressing Cyclin D1 (wild-type or K112E) or the empty vector (EV) that were nucleofected with RNPs containing Cas9 and the indicated sgRNAs and then treated with 2 µM PT2399 or DMSO for 4 days.

C, Cellular proliferation assays of cells as in (**A**) that were treated with 2 μM PT2399 or DMSO for 16 days. Data are means ± SD of n = 3 biological replicates and were normalized to the DMSO-treated cells for the respective CRISPRa sgRNAs. **, P < 0.01, ***, P < 0.001, and NS, Unpaired t test. **D,** Cellular proliferation assays of cells as in (**B**) that were treated with 2 μM PT2399 or DMSO for 16 days. Data are means ± SD of n = 3 biological replicates and were normalized to the DMSO-treated cells for the respective cell lines (EV, WT, or KE). **, P < 0.01, and NS, Unpaired t test.
