# Supplementary material for: Requirement for Cyclin D1 Underlies Cell-Autonomous HIF2 Dependence in Kidney Cancer
Source: Cancer Discov. 2025 Apr 4;15(7):1484–504. doi: 10.1158/2159-8290.CD-24-1378 (PMC12223508; doi:10.1158/2159-8290.CD-24-1378)
Supplement: Shirole Fig. S12 — Fig. S12: Loss of All 3 pRB Paralogs is Required to Confer Resistance to CDK4/6 Inhibitor [file cd-24-1378_shirole_fig.s12_suppsf12.pdf]

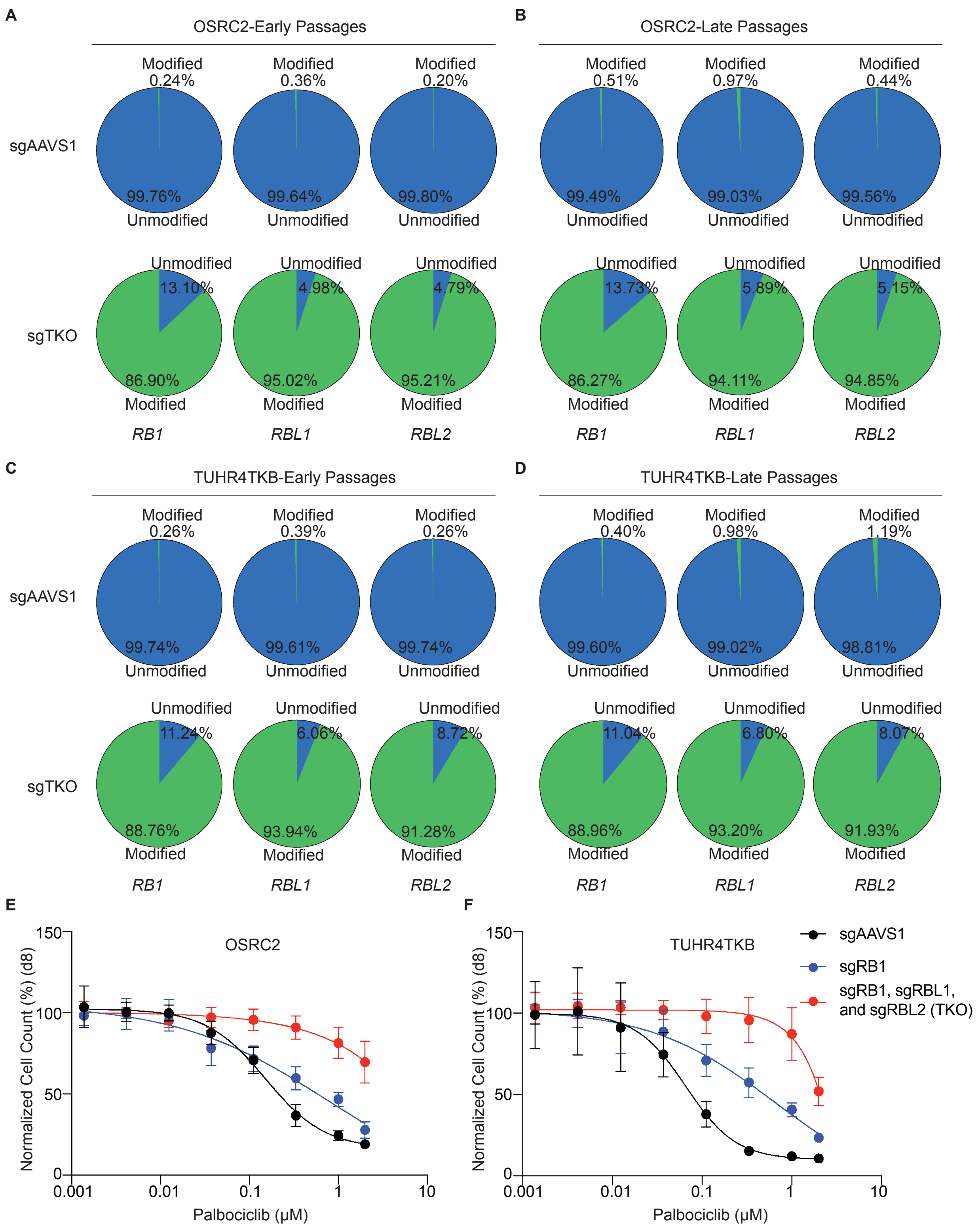
A
OSRC2-Early Passages
B
OSRC2-Late Passages
sgAAVS1
sgTKO
Modified 0.24%
99.76% Unmodified
Modified 0.36%
99.64% Unmodified
Modified 0.20%
99.80% Unmodified
Modified 0.51%
99.49% Unmodified
Modified 0.97%
99.03% Unmodified
Modified 0.44%
99.56% Unmodified
Unmodified 13.10%
86.90% Modified
Unmodified 4.98%
95.02% Modified
Unmodified 4.79%
95.21% Modified
Unmodified 13.73%
86.27% Modified
Unmodified 5.89%
94.11% Modified
Unmodified 5.15%
94.85% Modified
RB1
RBL1
RBL2
C
TUHR4TKB-Early Passages
D
TUHR4TKB-Late Passages
Modified 0.26%
99.74% Unmodified
Modified 0.39%
99.61% Unmodified
Modified 0.26%
99.74% Unmodified
Modified 0.40%
99.60% Unmodified
Modified 0.98%
99.02% Unmodified
Modified 1.19%
98.81% Unmodified
Unmodified 11.24%
88.76% Modified
Unmodified 6.06%
93.94% Modified
Unmodified 8.72%
91.28% Modified
Unmodified 11.04%
88.96% Modified
Unmodified 6.80%
93.20% Modified
Unmodified 8.07%
91.93% Modified
E
OSRC2
F
TUHR4TKB
Normalized Cell Count (%) (d8)
Palbociclib (μM)
0.001
0.01
0.1
1
10
0
50
100
150
sgAAVS1
sgRB1
sgRB1, sgRBL1, and sgRBL2 (TKO)

Fig. S12: Loss of All 3 pRB Paralogs is Required to Confer Resistance to CDK4/6 Inhibitor

A and B, NGS analysis of genomic DNA from OSRC2 early passages (**A**), late passages (**B**), cells that were nucleofected with RNPs containing Cas9 and the indicated sgAAVS1 or sgTKO (sgRB1, sgRBL1, and sgRBL2). The pie charts show the percentages of NGS reads that were modified (edited) or unmodified (not edited) at the *RB1*, *RBL1*, and *RBL2* genomic loci in the indicated cell lines. **C and D,** NGS analysis of genomic DNA from TUHR4TKB early passages (**C**), late passages (**D**), cells that were nucleofected with RNPs containing Cas9 and the indicated sgAAVS1 or sgTKO (sgRB1, sgRBL1, and sgRBL2). The pie charts show the percentages of NGS reads that were modified (edited) or unmodified (not edited) at the *RB1*, *RBL1*, and *RBL2* genomic loci in the indicated cell lines. **E and F**, Drug curves of OSRC2 (**E**) and TUHR4TKB (**F**) cells that were nucleofected with RNPs containing Cas9 and sgAAVS1, sgRB1, or sgTKO (sgRB1, sgRBL1, and sgRBL2), and then treated with indicated concentrations of Palbociclib for 8 days.
