# Supplementary material for: Requirement for Cyclin D1 Underlies Cell-Autonomous HIF2 Dependence in Kidney Cancer
Source: Cancer Discov. 2025 Apr 4;15(7):1484–504. doi: 10.1158/2159-8290.CD-24-1378 (PMC12223508; doi:10.1158/2159-8290.CD-24-1378)
Supplement: Shirole Fig. S13 — Fig. S13: Sustained Expression of CCND1 Alone is Not Sufficient to Confer Resistance to PT2399 In Vivo [file cd-24-1378_shirole_fig.s13_suppsf13.pdf]

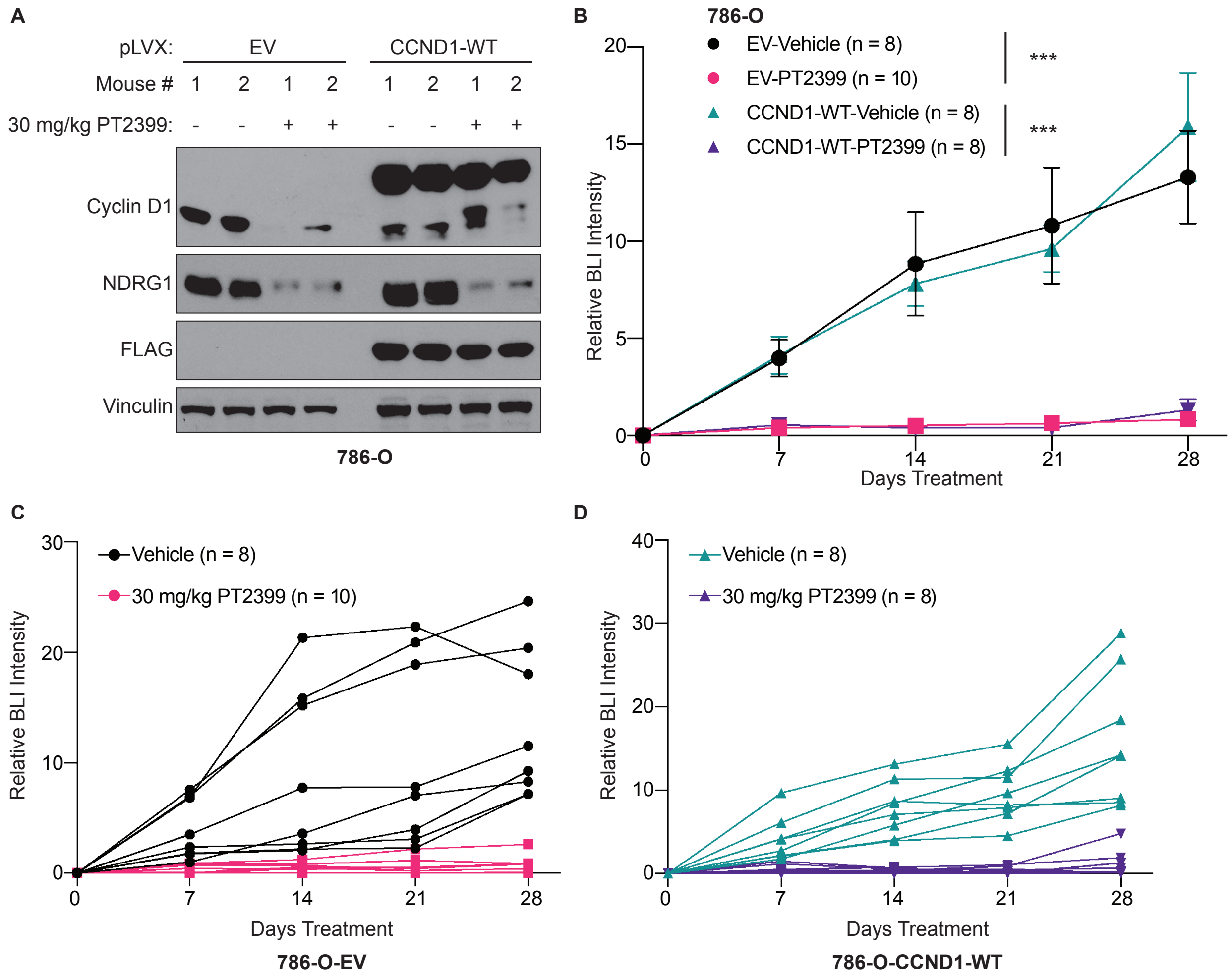

Fig. S13: Sustained Expression of *CCND1* Alone is Not Sufficient to Confer Resistance to PT2399 *In Vivo*

A, Immunoblot analysis of orthotopic xenografts formed by the 786-O cells that stably express firefly luciferase and either Cyclin D1 (wild-type) or the empty vector (EV) in mice that were treated with PT2399 (30 mg/kg) or vehicle daily for five days by oral gavage. **B,** Average relative BLI intensity over time of orthotopic xenografts formed by cells as in (**A**) that were treated with PT2399 (30 mg/kg) or vehicle daily for 28 days by oral gavage. For each mouse, BLI readings were normalized to the BLI value from the day the treatment was started. Data are mean ± SEM from three independent experiments. ***, P < 0.001 and NS, two-way Anova. **C and D,** Spider plots showing serial BLI values for the individual mice from (**B**).
