# Supplementary material for: Requirement for Cyclin D1 Underlies Cell-Autonomous HIF2 Dependence in Kidney Cancer
Source: Cancer Discov. 2025 Apr 4;15(7):1484–504. doi: 10.1158/2159-8290.CD-24-1378 (PMC12223508; doi:10.1158/2159-8290.CD-24-1378)
Supplement: Shirole Fig. S14 — Fig. S14: Failure to Downregulate VEGFA and CCND1 Confers Resistance to PT2399 In Vivo [file cd-24-1378_shirole_fig.s14_suppsf14.pdf]

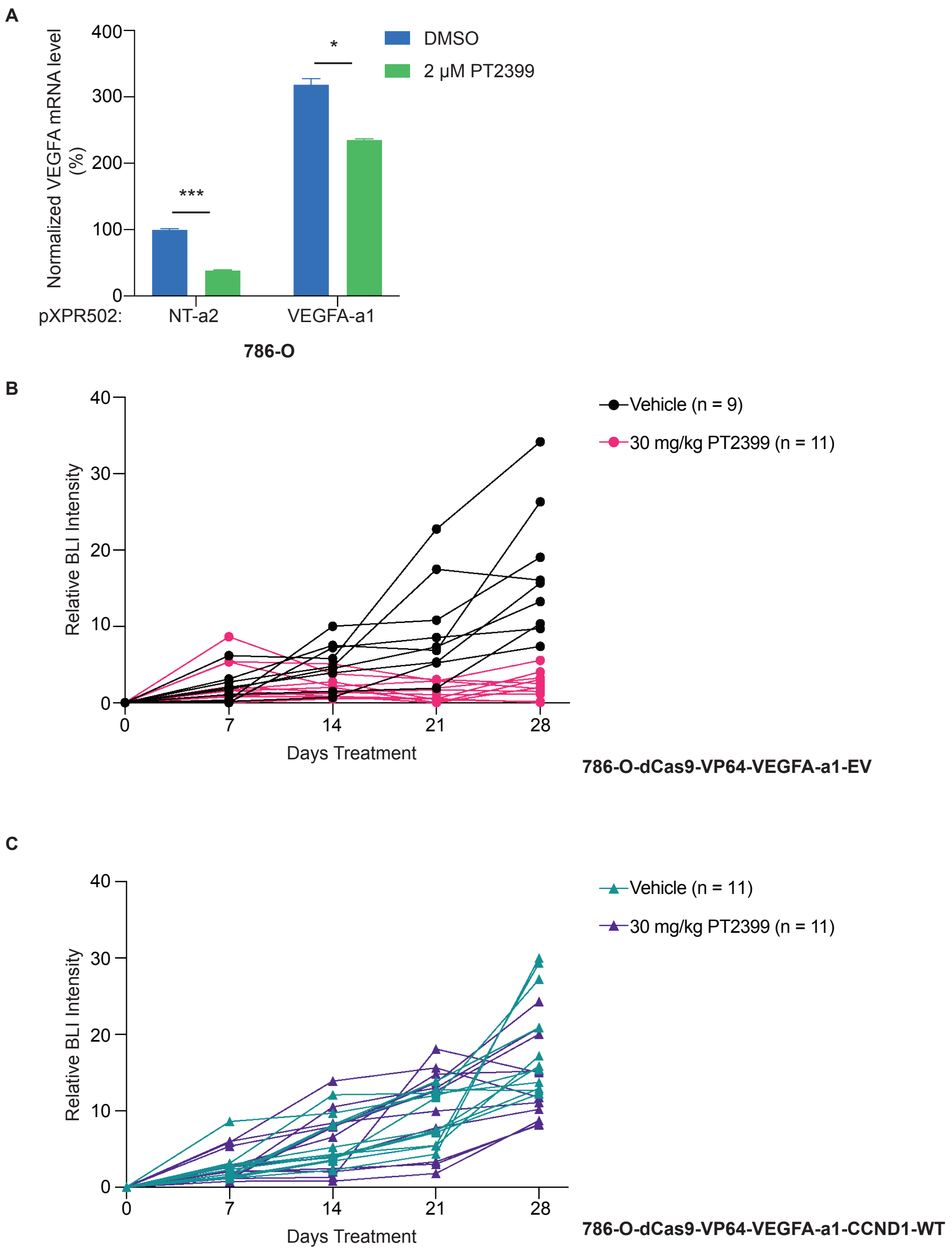

Fig. S14: Failure to Downregulate *VEGFA* and *CCND1* Confers Resistance to PT2399 *In Vivo*

A, *VEGFA* mRNA level from 786-O cells that stably express firefly luciferase, dCas9-VP64, and the indicated CRISPRa sgRNAs. The cells were treated with 2 μM PT2399 or DMSO for 48 hrs. The VEGF mRNA levels were normalized to the NT-a2 expressing

cells treated with DMSO. Data are means ± SD of n = 3 biological replicates. *, $P < 0.05$, and ***, $P < 0.001$, Unpaired t test. **B and C,** Spider plots showing serial BLI values for the individual mice from figure **6D**.
