# Supplementary material for: Requirement for Cyclin D1 Underlies Cell-Autonomous HIF2 Dependence in Kidney Cancer
Source: Cancer Discov. 2025 Apr 4;15(7):1484–504. doi: 10.1158/2159-8290.CD-24-1378 (PMC12223508; doi:10.1158/2159-8290.CD-24-1378)
Supplement: Shirole Fig. S15 — Fig. S15: High Cyclin D2 Expression as a Potential Cause of In Vitro HIF2-independence [file cd-24-1378_shirole_fig.s15_suppsf15.pdf]

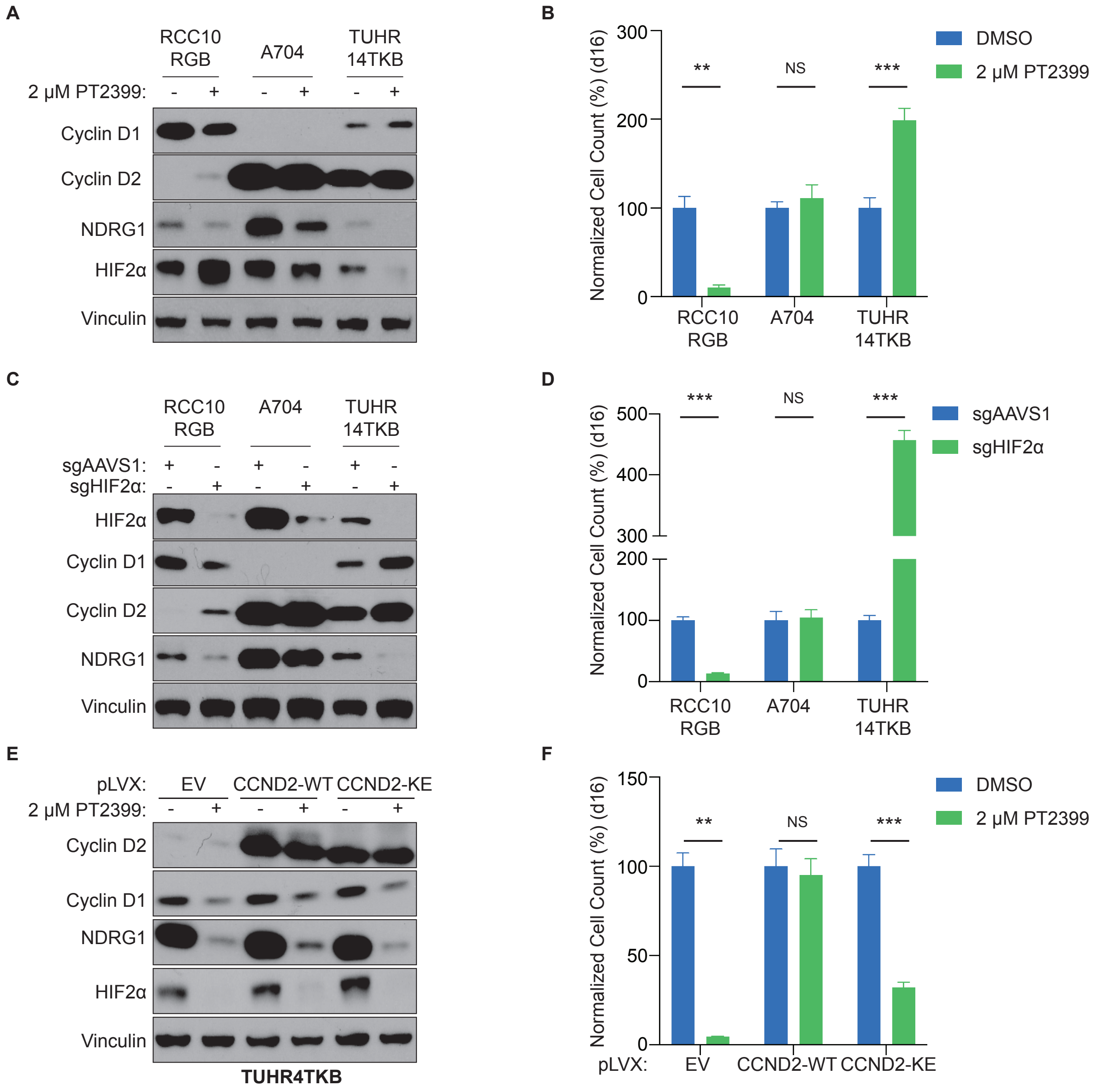


**Fig. S15: High Cyclin D2 Expression as a Potential Cause of *In Vitro* HIF2-independence**

**A,** Immunoblot analysis of indicated ccRCC cell lines that were treated with 2 μM PT2399 or DMSO for 4 days. **B,** Cellular proliferation assays of cells as in (**A**) that were treated with 2 μM PT2399 or DMSO for 16 days. Data are means ± SD of n = 3 biological replicates and were normalized to the DMSO-treated cells for the respective cell lines (RCC10RGB, A704, and TUHR14TKB). **, $P < 0.01$, ***, $P < 0.001$, and NS, Unpaired t test **C,** Immunoblot analysis of ccRCC cells lines that underwent CRISPR

editing with the indicated CRISPRko sgRNAs. **D,** Cellular proliferation assays of cells as in (**C**). Data are means ± SD of n = 3 biological replicates and are normalized to the sgAAVS1 cells for each of the respective cell lines (RCC10RGB, A704, and TUHR14TKB). ***, P < 0.001, and NS, Unpaired t test. **E,** Immunoblot analysis of TUHR4TKB cells stably expressing Cyclin D2 (wild-type or K111E) or the empty vector (EV) and treated with 2 μM PT2399 or DMSO for 4 days. **F,** Cellular proliferation assays of cells as in (**E**) that were treated with 2 μM PT2399 or DMSO for 16 days. Data are means ± SD of n = 3 biological replicates and were normalized to the DMSO-treated cells for the respective cell lines (EV, WT, or KE). **, P < 0.01, ***, P < 0.001, and NS, Unpaired t test.
